# Late-Stage Minimal Labeling of Peptides and Proteins for Real-Time Imaging of Cellular Trafficking

**DOI:** 10.1021/acscentsci.4c01249

**Published:** 2024-11-26

**Authors:** Ferran Nadal-Bufi, Raj V. Nithun, Fabio de Moliner, Xiaoxi Lin, Shaimaa Habiballah, Muhammad Jbara, Marc Vendrell

**Affiliations:** † Centre for Inflammation Research, The University of Edinburgh, EH16 4UU Edinburgh, U.K.; ‡ IRR Chemistry Hub, Institute for Regeneration and Repair, The University of Edinburgh, EH16 4UU Edinburgh, U.K.; § School of Chemistry, Tel Aviv University, 69978 Tel Aviv, Israel

## Abstract

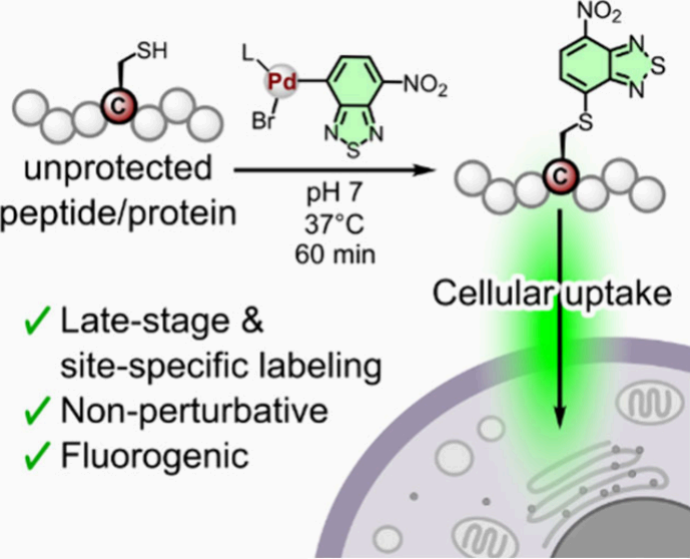

The cellular uptake routes of peptides and proteins are complex and diverse, often handicapping therapeutic success. Understanding their mechanisms of internalization requires chemical derivatization with approaches that are compatible with wash-free and real-time imaging. In this work, we developed a new late-stage labeling strategy for unprotected peptides and proteins, which retains their biological activity while enabling live-cell imaging of uptake and intracellular trafficking. Benzo-2,1,3-thiadiazoles were selectively incorporated into Cys residues of both linear and cyclic peptides via Pd-mediated arylation with good yields and high purities. The resulting labeled peptides are chemically stable under physiological conditions and display strong fluorogenic character for wash-free imaging studies. We utilized this approach to prepare native-like analogues of cell-penetrating peptides and performed time-course analysis of their internalization routes in live cells by fluorescence lifetime imaging. Furthermore, we applied our strategy to label the chemokine protein mCCL2 and monitor its internalization via receptor-mediated endocytosis in live macrophages. This study provides a straightforward strategy for late-stage fluorogenic labeling of intact peptides and small proteins and direct visualization of dynamic intracellular events.

## Abstract 2

Late-stage labeling to prepare unprotected fluorogenic peptides/proteins via Cys arylation with minimal bioactivity perturbation and suitable optical properties for wash-free imaging in live cells.

## Introduction

Targeted delivery of peptides and proteins into intracellular compartments remains a major challenge in macromolecule therapeutics because of the complexity and diversity of cellular uptake mechanisms. For cell-penetrating peptides (CPPs),^1−5^ different models of direct translocation have been proposed,^6−8^ which include carpet-like membrane disruption, the formation of transient pores or inverted micelles. CPPs can enter cells via endocytosis, an energy-dependent process that involves membrane binding, endosomal uptake and endosomal release.^9,10^ On the other hand, small proteins (e.g., chemokines) can enter cells via receptor-mediated endocytosis, a process that requires binding to membrane receptors for active internalization. In order to enhance our understanding of cell internalization pathways and trafficking of peptides and small proteins in live cells, fluorescence microscopy studies require labeling strategies that preserve their main physicochemical features while incorporating suitable reporters for real-time imaging.^11^

The derivatization of peptidic structures including CPPs and small proteins with commercial fluorophores has been used to investigate cell uptake and trafficking.^12−14^ Many fluorophores are based on relatively hydrophobic or charged structures^15,16^ that can alter the secondary structure and internalization mechanisms of native sequences,^17,18^ thus leading to inaccurate observations when studying macromolecule internalization.^19^ Other assays consider chemical tags that can be detected by interaction with secondary components (e.g., HaloTag), which have been described to quantify the cytosolic delivery of CPPs.^20^ However, this approach requires genetically modified cell lines and cannot provide mechanistic insights into the internalization routes of peptides or proteins.

**Figure 1:**
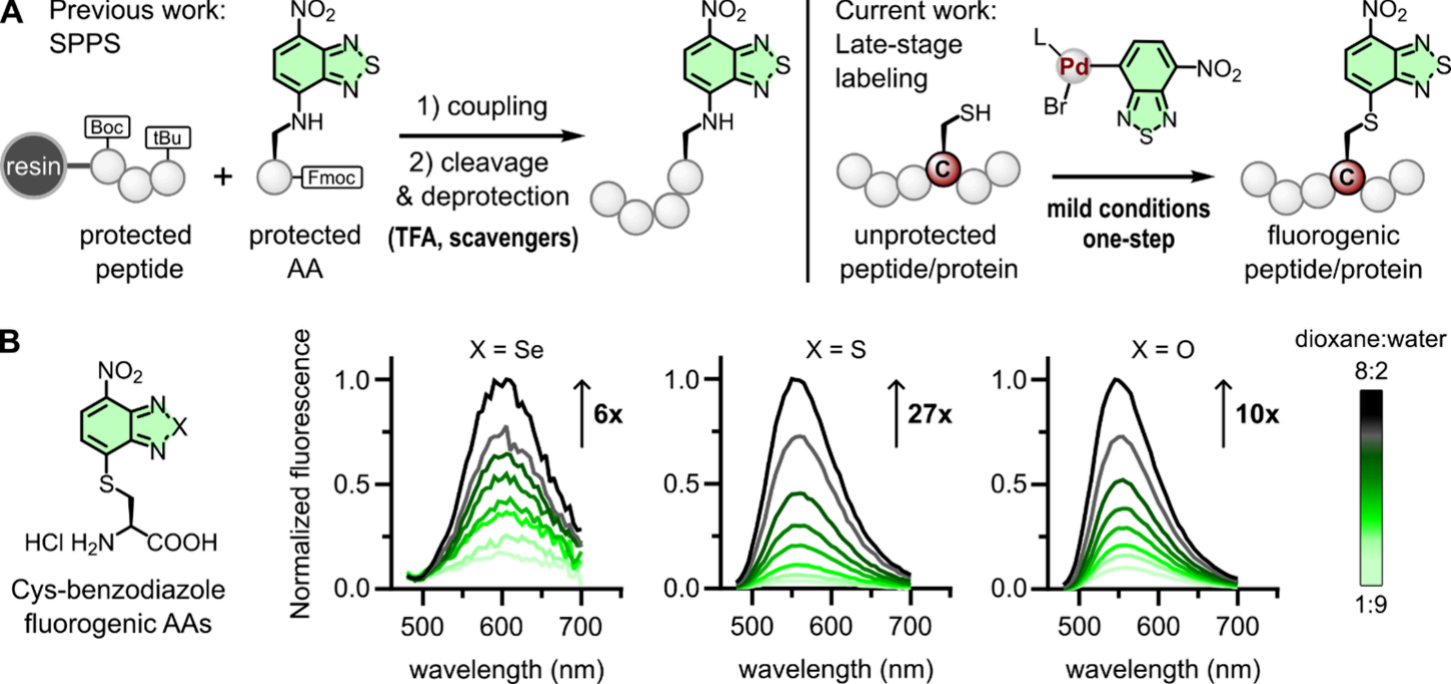
**Chemical strategies to synthesize fluorogenic peptides**. A) Left: previous work using benzodiazole amino acids for SPPS of fluorogenic peptides; right: late-stage and direct incorporation of benzodiazoles to unprotected peptides and small proteins. B) Representative normalized fluorescence emission spectra of Cys-based amino acids containing benzo-2,1,3-selenadiazole, benzo-2,1,3-thiadiazole and benzo-2,1,3-oxadiazole cores. Spectra of all compounds (10 μM) were recorded in dioxane:water mixtures after excitation at 460 nm. Fluorescence-fold increase ratios between hydrophobic and hydrophilic environments were determined by relating the emission intensities of each fluorophore in high (8:2) and low (1:9) ratios of dioxane:water mixtures.

Small fluorescent amino acids have emerged as promising building blocks for noninvasive labeling of peptides and proteins.^21−24^ In particular, fluorogenic amino acids are suitable for imaging dynamic events (e.g., interactions with membranes, localization in subcellular organelles) because they provide distinct fluorescence readouts in response to their microenvironments.^25−28^ Our group has reported benzodiazole-based amino acids for solid-phase peptide synthesis (SPPS) and demonstrated their application in fluorescence imaging.^29,30^ One major limitation of the SPPS of fluorogenic peptides is the potential degradation of fluorophores during synthetic steps (e.g., cleavage and removal of protecting groups) as well as their incompatibility with unprotected peptides, such as those obtained by recombinant methods. On the other hand, the incorporation of fluorogenic entities in proteins is largely limited to genetically encoded unnatural amino acids.^28,31^ In this work, we describe a novel late-stage and site-selective functionalization strategy to prepare native-like fluorescent analogues of CPPs and small proteins for real-time imaging of cellular trafficking (Figure 1A).

The orthogonal reactivity of cysteines (Cys) under physiological conditions make them convenient residues for direct diversification of biomolecules.^32−35^ Of particular interest, Cys can be selectively modified with aryl moieties to functionalize peptides with a broad range of chemical groups through stable S–C(sp2) bonds.^36,37^ Among these reactions, Pd-mediated C–S cross-couplings and Pd(II) oxidative addition complexes (Pd-OACs) are of particular interest due to their chemoselectivity, high reactivity and compatibility with mild reaction conditions.^38−41^ In view of these properties, we envisioned that the design of Pd-OACs bearing small benzodiazole fluorogens could be employed for site-selective and minimal labeling of complex biomolecules. The late-stage labeling with benzodiazole fluorogens provides important advantages, including 1) the small size and neutral character of these fluorophores ensures minimal perturbation to preserve native cellular uptake, 2) their fluorogenic character enables both wash-free and fluorescence lifetime imaging to feature the localization of peptides and proteins in subcellular structures (e.g., cell membrane, endosomes), and 3) the late-stage transfer of benzodiazole fluorophores via Cys-arylation results in chemically stable products.

## Results and Discussion

### Pd-SNBD Enables Fluorogenic, Selective, and Stable Labeling of Peptides

Benzodiazole fluorophores can be synthesized with variable bridging groups (e.g., oxygen, sulfur, selenium),^42^ therefore we first investigated the effect of different heteroatoms in the fluorogenic behavior of Cys-ligated constructs. We synthesized three Cys-benzodiazole amino acids including benzo-2,1,3-oxadiazole (**Cys-ONBD**), benzo-2,1,3-thiadiazole (**Cys-SNBD**) and benzo-2,1,3-selenadiazole (**Cys-SeNBD**) fluorophores, and measured their fluorescence properties in mixtures of dioxane:water, mimicking both hydrophilic and hydrophobic microenvironments. All three amino acids showed notable fluorogenic behavior (from 6-fold to 27-fold fluorescence increases, Figure 1B) with **Cys-SNBD** showing the lowest background signals in water and the strongest fluorescence response in hydrophobic conditions. The environmental sensitivity of **Cys-SNBD** was further corroborated by measuring the fluorescence emission in solvents with variable polarity (Figure S1), thus confirming the suitability of the benzo-2,1,3-thiadiazole core to build some of the first Pd-based fluorogenic reagents for late-stage incorporation in intact peptides and proteins (Figure 1A).

**Figure 2:**
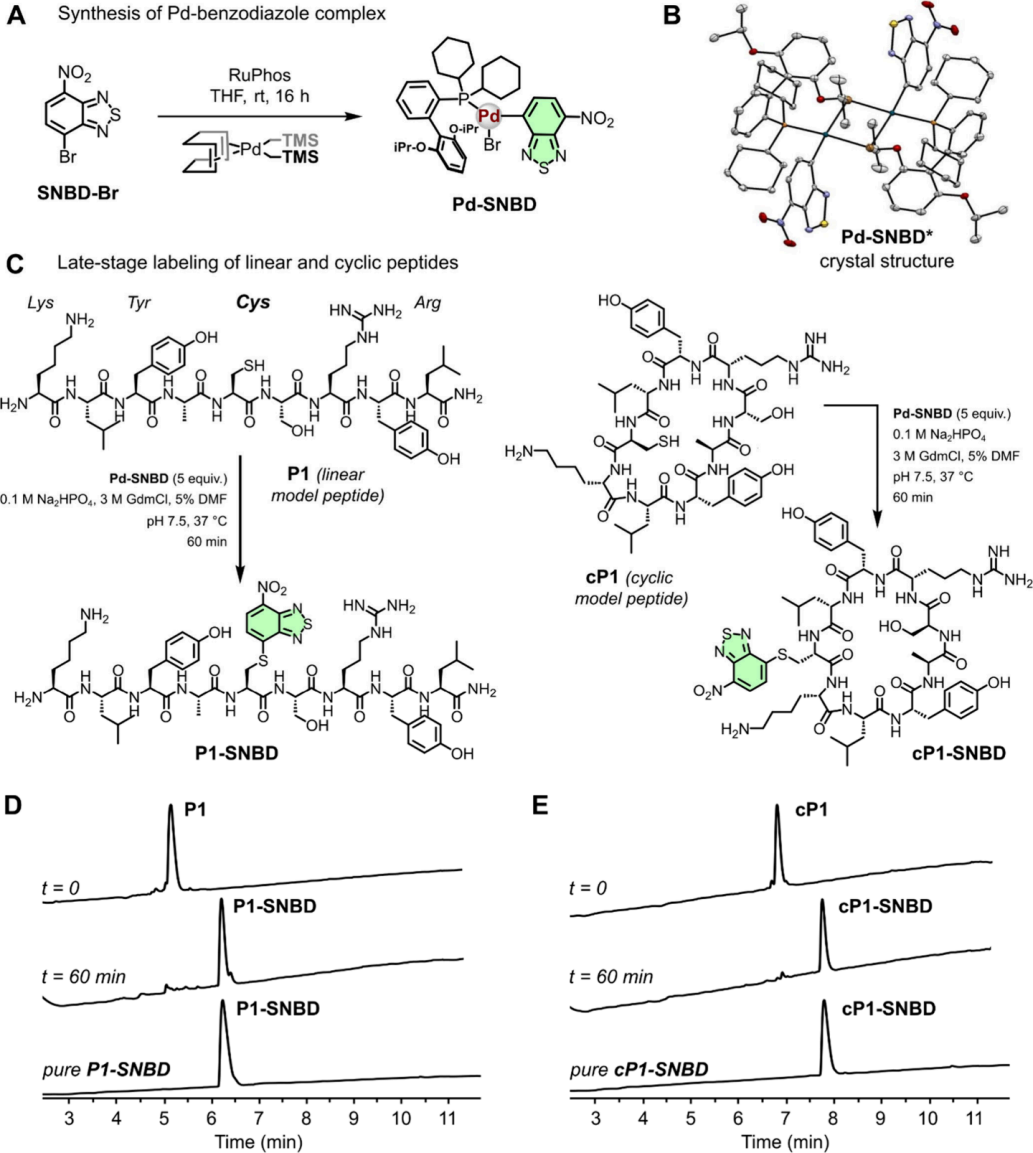
**Site-selective fluorogenic labeling of unprotected peptides by Pd-mediated S-arylation.** A) Chemical synthesis of the fluorogenic **Pd-SNBD** complex. B) Representation of the X-ray crystal structure of Pd-SNBD* (obtained as a dimer) [CCDC: 2374052]. C) Synthetic scheme for the site-specific labeling of the linear model peptide **P1** and the cyclic model peptide **cP1**. D, E) HPLC traces of the unprotected peptides **P1** and **cP1** and the resulting crude mixtures and isolated pure products after reaction with **Pd-SNBD** using the conditions detailed in panel C.

Encouraged by these results, we designed our labeling strategy by preparing one Pd-OAC bearing the benzo-2,1,3-thiadiazole group (**Pd-SNBD**) that would be suitable for reacting with Cys residues. For this purpose, we synthesized the brominated analogue **SNBD-Br** (Figure 2A) and coupled it to the complex [(COD)Pd(CH_2_TMS)_2_] in the presence of a RuPhos ligand^38^ to isolate (72% yield) and characterize the crystal structure of the desired **Pd-SNBD** complex (Figures 2A and 2B; see synthetic and full characterization details in Supporting Information). Notably, the synthesis of **Pd-SNBD** was scalable to 50 mg and stability studies proved that the reagent was stable and reactive after storage under nitrogen atmosphere and room temperature for several months (Figure S2).

We examined the site-selective reactivity of **Pd-SNBD** by synthesizing the linear model nonapeptide **P1** (sequence: KLYACSRYL, Figure 2C), which included one Cys residue as well as other reactive amino acids (e.g., Lys, Arg, Tyr). The unprotected peptide **P1** was synthesized using standard Fmoc/^t^Bu SPPS^43−45^ and subsequently coupled to **Pd-SNBD** under different reaction conditions, including variable temperatures, buffers and molar ratios (Figure S3). Time-course HPLC analysis of the coupling reactions confirmed the effective installation of the benzodiazole fluorogen onto the peptide **P1** using 5 equiv of **Pd-SNBD** in aqueous buffers and DMF as a cosolvent, and the Cys-selectivity was validated by LC-MS/MS (Figure S4). The control peptide **tP1**, which was an analogue of **P1** with Ala instead of Cys (sequence: KLYAASRYL), showed no labeling under the same experimental conditions, thus confirming the reactivity of **Pd-SNBD** with Cys (Figure S5). Remarkably, the optimization of the reaction conditions led to the clean formation of the linear fluorogenic peptide **P1-SNBD** as well as its head-to-tail cyclic analogue **cP1-SNBD** (Figure 2C) in less than 60 min with good conversions (e.g., 50–90%) and purities >90% after HPLC purification (Figures 2D and 2E).^46^ These findings confirmed the applicability of the **Pd-SNBD** complex as a straightforward fluorogenic reagent to effectively label Cys residues in unprotected linear and cyclic peptides.

Next, we investigated the optical properties and stability of the S-aryl linkage in the two labeled peptides **P1-SNBD** and **cP1-SNBD**. We examined their chemical stability after incubation in phosphate buffered saline (PBS, pH 7.0) at 25 °C and cell culture media at 37 °C (Figures S6 and S7) as well as different biologically relevant reducing environments (Figure S8). Notably, we found no major degradation in any of these conditions. Finally, we measured the emission spectra of **P1-SNBD** and **cP1-SNBD** and confirmed that the two peptides retained the fluorogenic behavior of benzodiazoles (Figure S9). Altogether, these results indicate that Pd-mediated S-arylation provides a rapid means to site-specifically label unprotected peptides with good yields and purities, and that SNBD-labeled peptides exhibit suitable properties for live-cell imaging under physiological conditions.

### SNBD-Labeled Peptides Maintain Secondary Structure and Physicochemical Properties of Peptides

**Figure 3:**
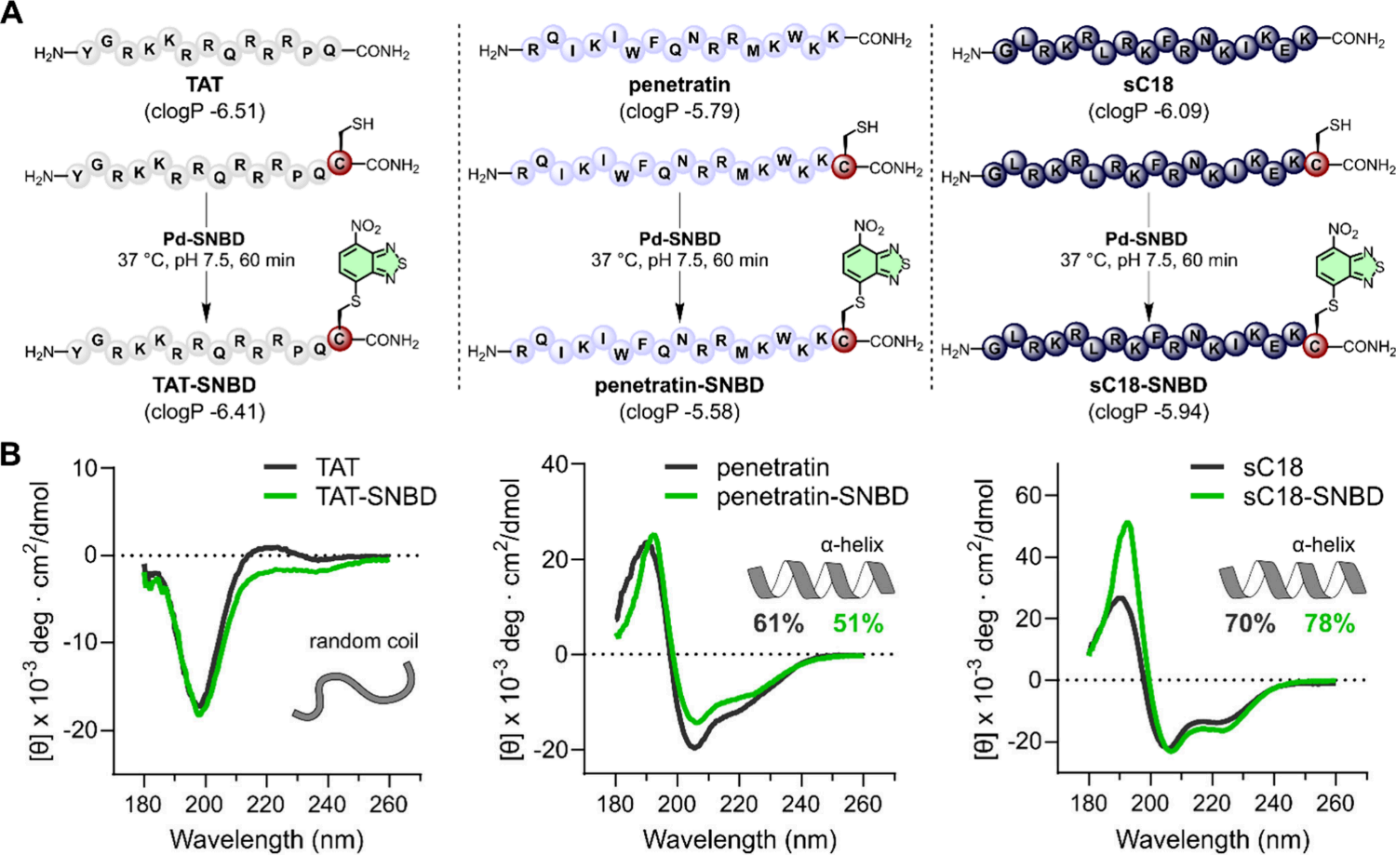
**Late-stage labeling of fluorogenic CPPs with minimal perturbation of physicochemical properties and secondary structure**. A) Late-stage labeling of the CPPs TAT, penetratin, and sC18 using the **Pd-SNBD** complex. Reaction conditions: 250 μM peptide were reacted with **Pd-SNBD** (1.25 mM) for 60 min followed by 3-MPA quenching and HPLC purification. Calculated logP (clogP) values for both unlabeled and SNBD-labeled peptides were calculated using the software Molinspiration. B) Representative CD spectra of unlabeled TAT, penetratin, and sC18 (black) and their SNBD-labeled counterparts (green). Spectra were recorded with 50 μM peptide in 30% trifluoroethanol in aqueous buffer (100 mM NaF, 10 mM KH_2_PO_4_ pH 7.5), and obtained at 0.5 nm intervals between wavelengths of 180 to 260 nm and averaged from 3 scans. Background signals were subtracted and the signals were recorded as millidegrees at 25 °C. Mean residue ellipticity (MRE) was calculated according to [θ]_MRE_ = θ/(c × l × N_r_), where θ is the recorded ellipticity in millidegrees, c is the peptide concentration in dmol·L^–1^, l is the cell path length in cm, and N_r_ is the number of residues. Cartoon representations of the secondary structures indicated by the CD spectra are depicted in gray, alongside the percentage of helicity (when applicable) for the unlabeled CPPs (gray) and labeled CPPs (green). The helical percentage (*H*_α_) was calculated from the Luo-Baldwin formula.^52^

Having proved the synthetic feasibility of late-stage benzodiazole incorporation in intact peptides, we explored the production of CPP derivatives for fluorescence imaging studies. Specifically, we prepared fluorogenic analogues of three reported sequences with different internalization mechanisms (i.e., TAT, penetratin and sC18, Figure 3A).^47−49^ The three peptides were prepared by SPPS including a C-terminal Cys residue for late-stage labeling and were isolated in yields around 50% and with purities >90% after HPLC purification (see full synthetic and characterization details in Supporting Information). Next, we reacted the three CPPs with the **Pd-SNBD** complex in 20 mM Tris buffer (pH 7.5) using DMF as a cosolvent at 37 °C. In all cases, we isolated the SNBD-labeled peptides as the main products (i.e., **TAT-SNBD**, **penetratin-SNBD** and **sC18-SNBD**, Figure 3A) and with recovery yields around 30%. Notably, we did not observe any cross-reactivity with other polar amino acids (i.e., Arg, Asn, Gln, Glu, Lys, Met) or aromatic amino acids (i.e., Trp and Tyr), and we confirmed the purity and identity of all analogues by HPLC-MS analysis (see full synthetic and characterization details in Supporting Information). Furthermore, we analyzed the potential presence of Pd impurities in purified **penetratin-SNBD** by inductively coupled plasma mass spectrometry (ICP-MS) and found levels under 15 ppm, which are compatible with biological assays under physiological conditions.^50^

Next, we studied the impact of SNBD fluorogenic labeling in the physicochemical and bioactivity properties of the three CPPs. We confirmed minimal differences between the clogP values of all unlabeled vs labeled peptides (Figure 3A) and compared their secondary structures by circular dichroism (CD) spectroscopy (Figure 3B). In these experiments, unlabeled TAT showed a CD spectrum with a negative minimum at ∼190 nm, which is characteristic of random coil structures. This profile was also observed for **TAT-SNBD**, suggesting that both peptides are unstructured in solution. The CD spectra of penetratin and **penetratin-SNBD** were both indicative of the presence of α-helical structures, with the characteristic mean residual ellipticity (MRE) minima at 208 and 222 nm. The calculated helicities were 61% for penetratin and 51% for **penetratin-SNBD**. The unlabeled sC18 peptide and its fluorogenic analogue **sC18-SNBD** also showed profiles indicative of α-helical structures with calculated helicities of 70% and 78%, respectively. These results suggest that the incorporation of SNBD fluorophores did not substantially alter the secondary structure of the CPPs, which is critical for their cell internalization mechanisms. We also evaluated the biological properties of all peptides by performing hemolysis assays as a bioactivity feature of most CPPs.^51^ The three fluorogenic **TAT-SNBD**, **penetratin-SNBD**, and **sC18-SNBD** peptides showed hemolytic activities in the same range as their unlabeled counterparts (i.e., EC_50_ values for the different peptides: TAT = 79 ± 6 μM and **TAT-SNBD** = 53 ± 2 μM; penetratin = 34 ± 5 μM and **penetratin-SNBD** = 27 ± 3 μM; sC18 = 70 ± 4 μM and **sC18-SNBD** = 43 ± 3 μM) (Figure S10). Overall, these results indicated that the physiochemical properties of CPPs were largely retained after labeling with benzo-2,1,3-thiadiazole, confirming that this strategy provides a suitable means to directly visualize the cellular uptake and trafficking of native-like peptide structures.

### Wash-Free Imaging of CPP Trafficking in Live Cells

**Figure 4:**
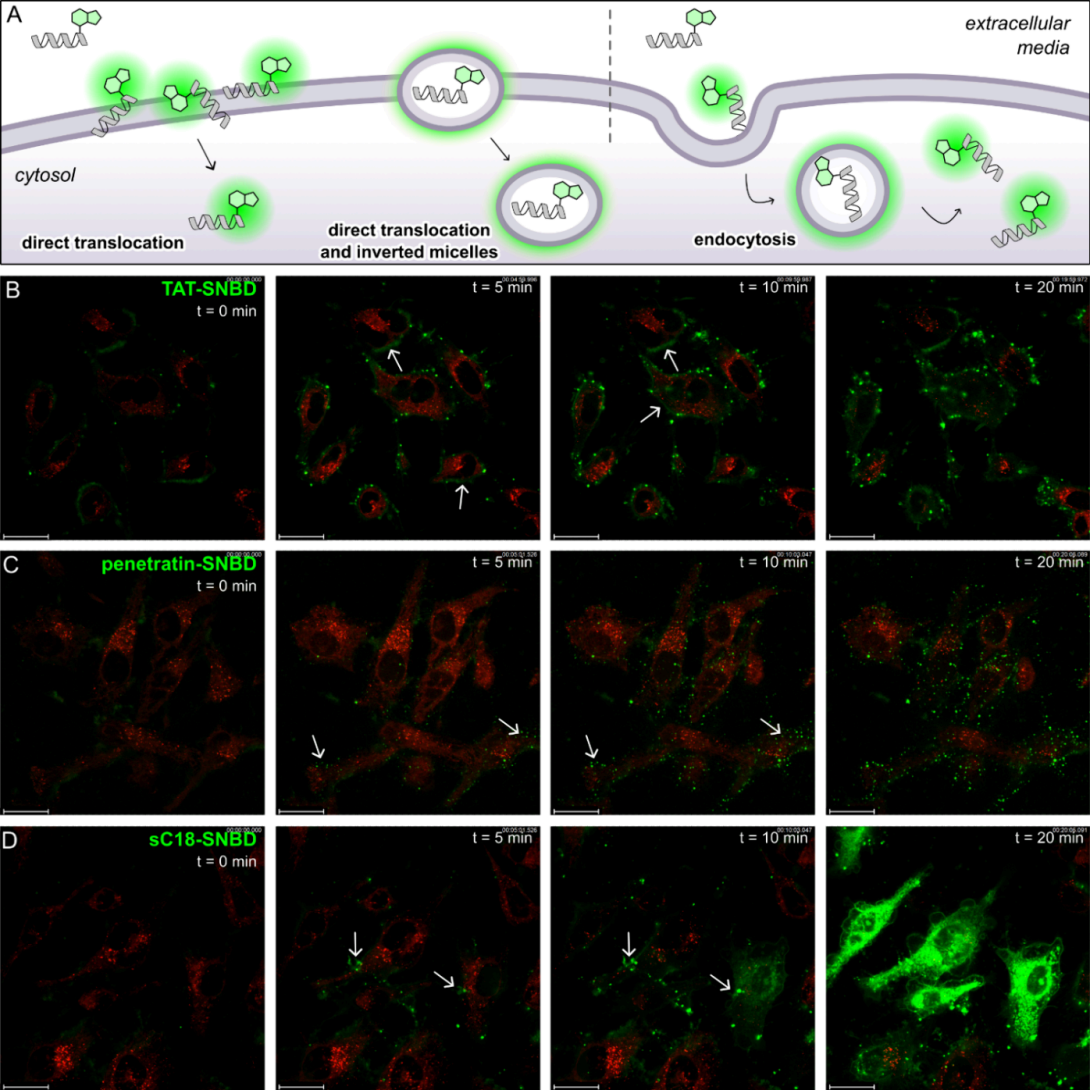
**Fluorescence imaging of CPP internalization and trafficking in live cells**. A) Cartoon depicting mechanisms of CPP internalization (from left to right): direct translocation, formation of inverted micelles, and endocytosis. B-D) Representative fluorescence microscopy images of HeLa cells after incubation with SNBD-labeled CPPs (10 μM) and imaged immediately by confocal microscopy (exc/em: 405/515 nm for SNBD-labeled CPPs and 561/592 nm for LysoTracker Red). Images display four relevant time points and movies for **TAT-SNBD** (Movie S1), **penetratin-SNBD** (Movie S2) and **sC18-SNBD** (Movie S3) are included in the Supporting Information. White arrows highlight membrane accumulation of **TAT-SNBD** in (A), intracellular inverted micelles of **penetratin-SNBD** in (B), and endosomal uptake of **sC18-SNBD** in (C). Scale bars: 25 μm.

Having synthesized native-like fluorogenic analogues of three different CPPs, we conducted wash-free microscopy experiments in live HeLa cells as a well-established line for imaging studies of cellular internalization.^53,54^ The three labeled CPPs were added to the cell medium, and their fluorescence localization was monitored over 20 min without any washing steps and using LysoTracker Red as a subcellular marker. **TAT-SNBD** showed internalization by direct translocation (Figure 4A) with high accumulation across the cell membrane, and, to a lesser degree, diffusion throughout the cytoplasm over time (Figure 4B, Figure S11 and Movie S1). This behavior was consistent with previously reported internalization studies showing direct translocation,^48^ where the high local concentration of TAT results in transient disruptions of the plasma membrane and subsequent cell entry. Furthermore, the dynamin inhibitor MiTMAB which impairs endocytosis,^55,56^ did not affect the distribution of **TAT-SNBD** (Figure S12), indicating that its internalization was energy-independent.

In contrast, **penetratin-SNBD** showed a punctate distribution indicative of vesicle accumulation (Figure 4A). Interestingly, these vesicles were not located in the membrane but internalized and distributed across the cytosol (Figure 4C and Movie S2), as confirmed by Z-stack imaging microscopy (Figure S13). This observation correlates with the inverted micelle mechanism that was first described for penetratin.^48^ Interestingly, the coadministration of **penetratin-SNBD** with MiTMAB reduced the number of fluorescent intracellular vesicles (Figure S14), suggesting that the internalization of penetratin can also partially occur via endocytosis as described in some literature reports.^48,57,58^

Lastly, the labeled peptide **sC18-SNBD** exhibited a typical endocytic mechanism of internalization (Figure 4A).^49,59^ After initial accumulation at the plasma membrane, **sC18-SNBD** showed clear intracellular punctate distribution (e.g., endosomes) and subsequently spread across the cells with high efficiency (Figure 4D, Figure S15 and Movie S3). In this case, the coadministration with MiTMAB did prevent the cell uptake to a large extent, thereby confirming that the internalization of **sC18-SNBD** was mediated by endocytosis (Figure S16). The results of these experiments indicate that late-stage SNBD labeling is a straightforward strategy for nonperturbative modification of unprotected peptides and corroborate its application for mechanistic studies of internalization and trafficking in three prototypical peptide entities.

**Figure 5:**
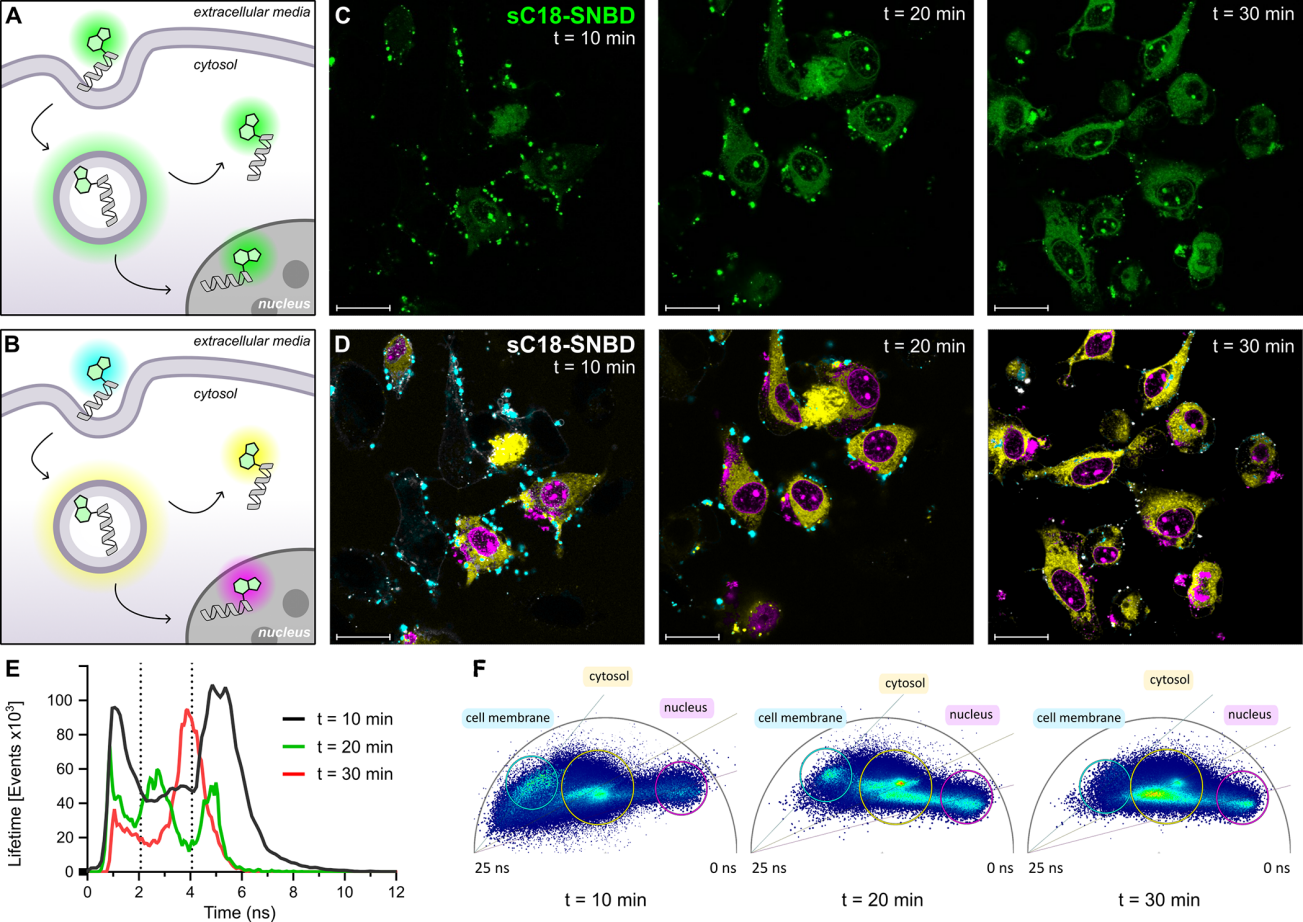
**FLIM of live HeLa cells and real-time trafficking of sC18-SNBD**. A-B) Cartoon depicting the mechanisms of **sC18-SNBD** internalization with pseudocolors highlighting fluorescence readouts using standard microscopy (A) or FLIM (B). C-D) Representative fluorescence microscopy images (C) and FLIM images (D) of HeLa cells after incubation with **sC18-SNBD** (10 μM) and immediate acquisition by confocal microscopy (exc/em: 440/515). Images display three relevant time points at 10, 20, and 30 min. FLIM movie (Movie S4) is included in the Supporting Information. E) Histogram plots of fluorescence lifetime events measured for representative images at 10 min (black), 20 min (green), and 30 min (red). F) Phasor plots and population gating for each of the images in (D). Harmony = 2. Scale bars: 25 μm.

To further increase the spatiotemporal resolution of cell internalization and trafficking, we explored the compatibility of the peptide **sC18-SNBD** for fluorescence lifetime imaging microscopy (FLIM) in HeLa cells. Given the fluorogenic character of SNBD, we envisaged that variations in its fluorescence lifetime would enable time-course monitoring of the subcellular localization of the CPP (Figures 5A and 5B). For these experiments, we incubated HeLa cells with **sC18-SNBD** and acquired real-time fluorescence intensity as well as FLIM images. Importantly, we confirmed that the FLIM readouts of **sC18-SNBD** were well above the intensity threshold of autofluorescence in unstained HeLa cells (Figure S17). Unlike fluorescence intensity readouts (Figure 5C), the FLIM analysis differentiated three clear lifetime components both in the microscope images (Figure 5D), histograms (Figure 5E), and in the phasor plots (Figure 5F), with the longest lifetimes (∼6 ns) corresponding to the initial interactions of **sC18-SNBD** with the plasma membrane, and shorter lifetimes observed upon internalization to the cytosol and translocation to the nucleus (Figure 5E). Importantly, the localization of **sC18-SNBD** in different subcellular compartments provided valuable insights into its mechanisms of internalization. For instance, we observed distinct nuclear localization of **sC18-SNBD** even at early time points (i.e., 10 min), which suggests that this CPP can undergo rapid direct translocation in addition to the reported gradual cellular endocytosis, a dual internalization mechanism that has been reported for some CPPs.^49,59,60^

Altogether, these results confirm that the environmental sensitivity of SNBD is optimal for FLIM imaging and represents a useful reporter to differentiate the complex pathways of CPP internalization with high spatiotemporal resolution.

### Fluorescence Labeling of Chemokine Proteins with Pd-SNBD

**Figure 6:**
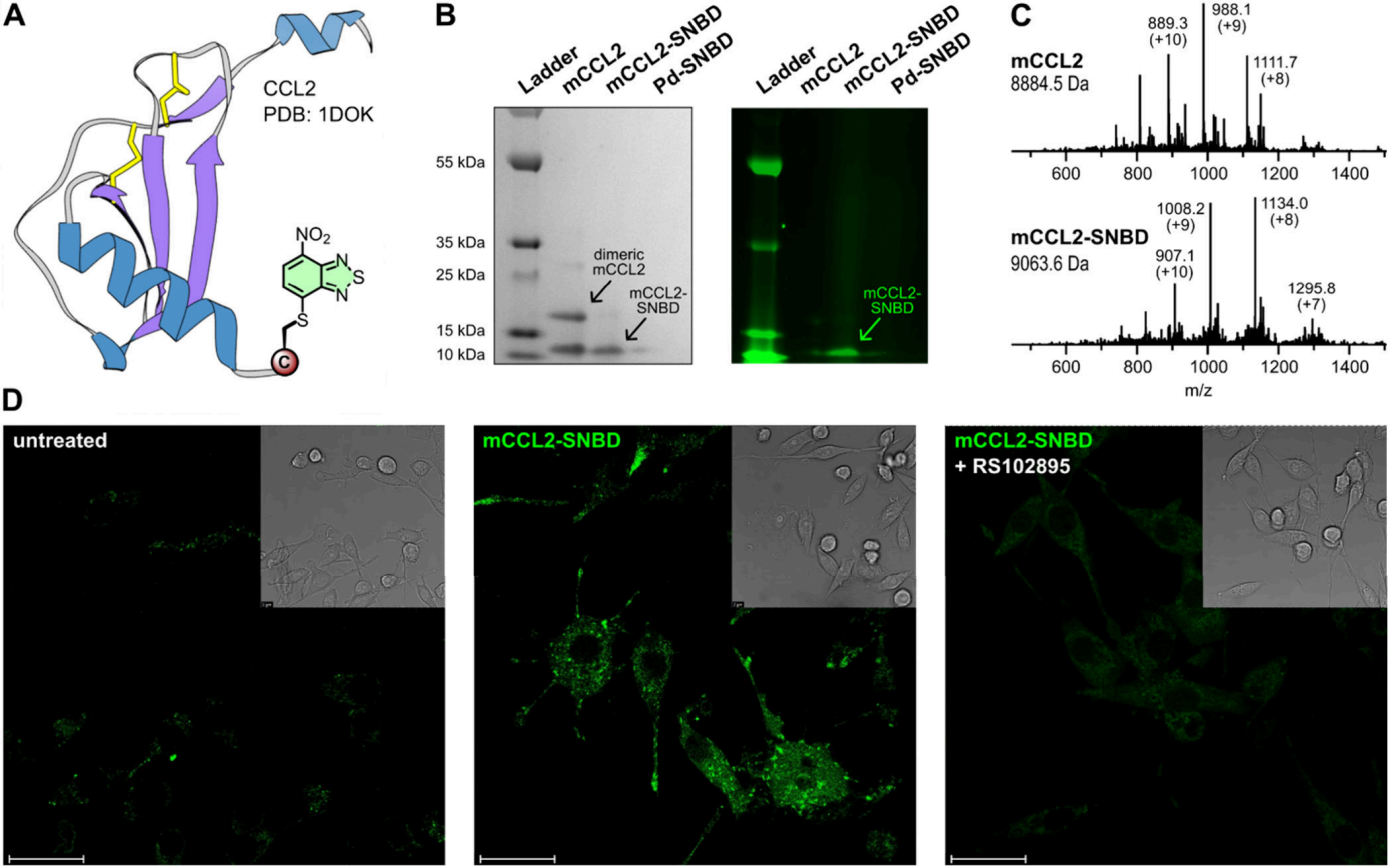
**Site-selective mCCL2 chemokine labeling and real-time imaging of receptor-mediated endocytosis in live macrophages.** A) Schematic representation of the chemokine mCCL2 (PDB: 1DOK) depicting the incorporation of SNBD at the C-terminal end. B) Coomassie-stained and in-gel fluorescence scanning of unlabeled mCCL2, **mCCL2-SNBD**, and the **Pd-SNBD** complex. C) Mass spectrometry analysis of unlabeled mCCL2 and **mCCL2-SNBD** proteins. D) Representative fluorescence confocal microscopy images of RAW264.7 cells after incubation with **mCCL2-SNBD** for 1 h. Left panel: untreated cells; middle panel: cells treated with **mCCL2-SNBD** (4 μM); right panel: cells treated with **mCCL2-SNBD** (4 μM) and the CCR2 antagonist RS102895 (40 μM) (exc/em: 405/515 nm). Insets show the corresponding brightfield images. Scale bars: 25 μm.

Next, we decided to expand the scope of our minimal labeling strategy to proteins and labeled the mouse chemokine ligand 2 (mCCL2). mCCL2 is a ∼10 kDa small protein that plays a major role in chemotaxis and has been associated with the recruitment of macrophages to the tumor microenvironment.^61−64^ mCCL2 binds to its cognate chemokine receptor CCR2 and enters macrophages via receptor-mediated endocytosis.^62^ For our studies, we employed the full-length mCCL2 with two disulfide bonds in the native sequence and an additional Cys residue at the C-terminal end for SNBD labeling (Figure 6A). Because the *N*-terminus of mCCL2 is important for receptor binding,^63^ we chose to incorporate SNBD at the C-terminal end. First, mCCL2-Cys (50 μM) was incubated with **Pd-SNBD** (5 equiv) under nonreducing conditions (20 mM Tris, pH 7.5, 5% DMF, 37 °C) for 30 min, after which the reaction was quenched with 3-mercaptopropionic acid and purified using a protein desalting column. We analyzed the labeled **mCCL2-SNBD** by SDS-PAGE under nonreducing conditions, which featured a single band at the correct molecular weight with strong in-gel fluorescence for **mCCL2-SNBD** (Figure 6B). Unlabeled mCCL2 showed an additional band corresponding to the dimer (∼20 kDa) formed via disulfide bond of the free C-terminal Cys residue,^64^ while the conjugation of Cys to SNBD prevented the formation of dimers in **mCCL2-SNBD.** Mass spectrometry analysis further confirmed equimolar labeling with the disulfide bonds of **mCCL2-SNBD** remaining unaffected (Figure 6C).

Next, we acquired fluorescence microscopy images in live RAW264.7 macrophages as a murine macrophage cell line expressing the chemokine receptor CCR2.^64^ We incubated the macrophages with **mCCL2-SNBD**, and fluorescence images were taken after 1 hour without any washing steps. RAW264.7 cells showed bright fluorescence signals distributed across the cytosol confirming internalization of **mCCL2-SNBD** (Figure 6D). Furthermore, we confirmed the receptor-mediated endocytosis of **mCCL2-SNBD** by performing the same microscopy experiments in the presence of the CCR2 antagonist RS102895,^65,66^ which led to substantially reduced intracellular fluorescence emission (Figure 6D). These results confirm that late-stage SNBD labeling is not only compatible with peptide structures but also with full-length proteins, thus opening new opportunities to study the internalization and localization of complex biomolecules (e.g., cytokines) in live cells.

## Conclusions

In summary, we have successfully developed a novel late-stage strategy to label fluorogenic peptides and proteins for real-time imaging of cellular trafficking. We have optimized the Cys-selective, Pd-mediated arylation of unprotected biomolecules with small benzodiazole fluorogens and demonstrated its utility for the rapid synthesis of linear and cyclic peptides with high yields and purities. Importantly, the resulting labeled peptides are stable under physiological conditions and show strong fluorogenic behavior in both intensity-based and FLIM measurements. We demonstrated the application of this labeling strategy in multiple CPPs (TAT, penetratin, and sC18) and one protein (mCCL2). SNBD-labeled biomolecules retained their physicochemical and bioactivity properties and were used in wash-free imaging experiments in live cells for in situ visualization of their uptake mechanisms, namely direct translocation, micelle-mediated internalization, and endocytic uptake. Furthermore, the FLIM analysis of the labeled CPP **sC18-SNBD** clearly detected its subcellular localization in the cell membrane, cytosol and nucleus, and indicated that this peptide can undergo direct nuclear translocation in live cells. Finally, we showed successful labeling and live-cell imaging of the chemokine mCCL2 in mouse macrophages, underscoring the applicability of this approach to a wide range of peptide and protein-based structures. This work constitutes an efficient strategy for direct fluorogenic labeling of intact peptides and proteins and holds potential for future studies of intracellular trafficking, drug delivery, and biological therapeutics.^67,68^

## Supporting Information Available

Detailed synthetic procedures and full chemical characterization data, methods, supplementary figures, NMR spectra (PDF)
Movie S1. Time-lapse fluorescence confocal microscopy images of HeLa cells after incubation with **TAT-SNBD** (10 μM, green) and the lysosomal counterstain LysoTracker (red) (AVI)
Movie S2. Time-lapse fluorescence confocal microscopy images of HeLa cells after incubation with **penetratin-SNBD** (10 μM, green) and the lysosomal counterstain LysoTracker (red) (AVI)
Movie S3. Time-lapse fluorescence confocal microscopy images of HeLa cells after incubation with **sC18-SNBD** (10 μM, green) and the lysosomal counterstain LysoTracker (red) (AVI)
Movie S4. Time-lapse FLIM images of HeLa cells after incubation with sC18-SNBD (10 μM) (AVI)
Transparent Peer Review report available (PDF)

## Supplementary Material

oc4c01249_si_001.pdf

oc4c01249_si_002.avi

oc4c01249_si_003.avi

oc4c01249_si_004.avi

oc4c01249_si_005.avi
