# Supplementary material for: Late-Stage Minimal Labeling of Peptides and Proteins for Real-Time Imaging of Cellular Trafficking
Source: ACS Cent Sci. 2024 Nov 26;11(1):66–75. doi: 10.1021/acscentsci.4c01249 (PMC11758221; doi:10.1021/acscentsci.4c01249)
Supplement: Supplementary file 1 — oc4c01249_si_001.pdf [file oc4c01249_si_001.pdf]

Electronic Supporting Information

Late-stage Minimal Labeling of Peptides and Proteins for Real-time Imaging of Cellular Trafficking

Ferran Nadal-Bufi,[a,b,¥] Raj V. Nithun,[c,¥] Fabio de Moliner,[a,b] Xiaoxi Lin,[c] Shaimaa Habiballah,[c] Muhammad Jbara,[c,*] Marc Vendrell[a,b,*]

[a] Centre for Inflammation Research, The University of Edinburgh, EH16 4UU Edinburgh, UK.

[b] IRR Chemistry Hub, Institute for Regeneration and Repair, The University of Edinburgh, EH16 4UU Edinburgh, UK.

[c] School of Chemistry, Tel Aviv University, 69978 Tel Aviv, Israel.

¥ These authors contributed equally to this work.

* Corresponding authors: Muhammad Jbara, e-mail: jbaram@tauex.tau.ac.il; Marc Vendrell, e-mail: marc.vendrell@ed.ac.uk.

Table of Contents

1. Experimental Details
2. Chemical Synthesis
3. Supplementary Figures
4. Movie Legends
5. NMR Spectra
6. Crystal Data and Structure Refinement
7. Supplementary References

1. Experimental Details

Materials and methods. 4,7-Dibromo-2,1,3-benzothiadiazole was purchased from Fluorochem. Concentrated HNO_3 was purchased Fisher Scientific. Fmoc-L-Phe-OH, Fmoc-L-Asn(Trt)-OH, Fmoc-L-Met-OH, Fmoc-L-Gln(Trt)-OH, Fmoc-L-Arg(Pbf)-OH, Fmoc-L-Tyr(tBu)-OH, Fmoc-L-Glu(OtBu)-OH, Fmoc-L-Ala-OH, Fmoc-L-Leu-OH, Fmoc-L-Pro-OH, Fmoc-L-Cys(Trt)-OH, Fmoc-L-Lys(Boc)-OH, Fmoc-L-Ile-OH, Fmoc-L-Ser(tBu)-OH, Fmoc-Gly-OH, Fmoc-Trp(Boc)-OH, deuterated $CDCl_3$ and RuPhos ligand were purchased from Sigma-Aldrich. (2-(1H-Benzotriazol-1-yl)-1,1,3,3-tetramethyluronium hexafluoro-phosphate (HBTU) and HOBt were purchased from Luxembourg Bio Technologies Ltd. 2-chlorotrityl chloride polysterene resin was obtained from Chem impex and Rink Amide ProTide resin was obtained from CEM. Diethyl ether (Et_2O, 99.8% stabilized, ACS grade) was obtained from MACRON. Dichloromethane (CH_2Cl_2, ≥99.5% stabilized with 50 ppm Amylene) was obtained from CHEM-LAB. Peptide Synthesis-grade N,N-dimethylformamide (DMF) was purchased from Fisher Scientific. Acetonitrile (ACN, LC/MS Grade) was purchased from J. T. Baker. Trifluoroacetic acid (TFA, ≥99% ReagentPlus®), diisopropylethylamine (DIEA, ≥99% ReagentPlus®), piperidine (≥99% ReagentPlus®), triisopropylsilane (TIS, 98%), formic acid (FA, 98-100% for LC/MS), n-pentane (AR) and dimethyl sulfoxide (DMSO, ≥99.5% ReagentPlus®) were purchased from Bio-Lab Ltd. Water for all reactions carried out on proteins and for reverse-phase purification was obtained via filtration of deionized water through a MilliporeSigma Milli-Q^{TM} Ultrapure Water System. Deuterated dichloromethane-d_2 99.8% was purchased from ZEOtope. All chemicals were used as received from the suppliers without further purification. mCCL2 protein with an additional

Cys residue at the C-terminal was purchased from Preprotech. Spectroscopic data was measured on a Synergy HT spectrophotometer (Biotek) and the data analysis was performed using GraphPad Prism 5.0. Analytical RP-HPLC chromatograms were acquired using Thermo Scientific Vanquish HPLC. Mobile phases used are solvent A (0.05% TFA in water) and solvent B (0.05% TFA in ACN). Mass spectra were obtained using Thermo Scientific ISQ EM Mass spectrometer. Preparative RP-HPLC purifications were performed using Thermo Scientific DIONEX UltiMate 3000 Variable Wavelength Detector, equipped with Jupiter® 5 µm C4 300 Å LC column (250 x 10 mm^2). HRMS (ESI positive) were obtained with a Bruker ESI Micro-TOF mass spectrometer. Mobile phases were solvent A (0.05% TFA in water), and solvent B (0.05% TFA in ACN).

Cell culture and imaging of CPPs. HeLa and RAW264.7 cells were grown in Dulbecco's modified Eagle's medium (DMEM) supplemented with 10% (v/v) fetal bovine serum (FBS), 20 mM L-glutamine, and 1% (v/v) penicillin–streptomycin. HeLa cells were resuspended in growth medium containing 10% (v/v) FBS, counted using a Countess II FL, plated (10,000 cells $well^{-1}$) in a µ-slide 18-well glass bottom chamber (IBIDI®), and incubated at 37 °C with 5% CO_2 for 24 h. Afterwards, the medium was removed by suction and replaced with 100 µL of phenol red-free medium containing Lysotracker red (50 nM) or not. Afterward, 10 µL of labeled CPPs or mCCL2 were added for a final concentration of 10 µM and 4 µM, respectively. Cells were imaged at 37 °C in a Leica SP8 fluorescence confocal microscope (405/515 nm) equipped with a live-cell imaging stage using a HC PL APO CS2 40x/1.30 oil lens. For time-lapse imagining, fluorescence and brightfield images were acquired immediately after addition of CPPs and every minute during 30 min.

Images were acquired and processed with the corresponding microscope software, Leica Application Suite X (LAS X) V1.4.6. For Z-stack imagining microscopy, 40-50 images were acquired across a physical length of 15-20 µm. For inhibition of endocytosis, MiTMAB™ (15 µM) was added into the cells and incubated during 15 min prior to the addition of labeled CPPs (10 µM). Endpoint images were acquired as previously described after incubation at 37 °C with 5% CO_2 for 30 min. For inhibition of mCCL2 internalization, RS102895 (40 µM) was added into the cells and incubated during 15 min prior to the addition of **mCCL2-SNBD** (4 µM). Endpoint images were acquired as previously described after incubation at 37 °C with 5% CO_2 for 1 h. Images were acquired and processed with the corresponding microscope software, Leica Application Suite X (LAS X) V1.4.6.

Circular dichroism spectroscopy. Spectra were acquired in a Chirascan VX (Applied Photophysics, UK) with 50 µM peptide in 30% trifluoroethanol in aqueous buffer (100 mM NaF, 10 mM KH_2PO_4 pH 7.5) at 0.5 nm intervals between wavelengths of 180 nm to 260 nm using a 0.4-mm-path length quartz cell. Data were averaged from 3 scans and background signals were subtracted. The signals were recorded as millidegrees at 25 °C, and the mean residue ellipticity (MRE) was calculated according to $[\theta]_{MRE} = \theta/(c \times l \times N_r)$, where θ is the recorded ellipticity in millidegrees, c is the peptide concentration in dmol·L^{-1}, l is the cell path length in cm, and N_r is the number of residues. The helical percentage (H_α) was calculated from the Luo-Baldwin formula.

cLogP calculations. cLogP values were calculated based on the simplified molecular-input line-entry systems (SMILES) of each compound using the software Molinspiration.

FLIM. HeLa cells were grown and treated with **sC18-SNBD** as described in *Cell culture and imaging of CPPs.* Afterward, intensity and lifetime images were acquired at 37 °C in a Leica SP8 FALCON confocal microscope (440/515 nm) equipped with a live-cell imaging stage using a HC PL APO CS2 40x/1.30 oil lens. Laser frequency was set at 40 mHz allowing a decay of 25 ns and signal was collected with a HyD detector in counting mode. Lifetime images were processed with the corresponding microscope software, Leica Application Suite X (LAS X) V1.4.6 using a n-exponential reconvolution model with three components. An intensity threshold of 20 counts per pixel was established to calculate the lifetime. FLIM-phasor plots were used to identify signal population with a harmony of 2.

Haemolysis assays. Human blood was collected from healthy donors and following the protocols approved by the Centre for Inflammation Research Blood Resource at the University of Edinburgh (EMREC Reference number 21-EMREC-041). Human red blood cells were separated from serum and washed in phosphate-buffered saline (PBS, pH 7.4) with 4–5 times centrifugation at 4000 rpm for 1 min. Erythrocytes were then resuspended in PBS as a 0.5% (v/v) solution, and co-incubated with serially diluted peptides in a 96-well plate for 1 h at 37 °C. The 96-well plate was centrifuged to pellet the intact red blood cells, and disruption of red blood cell membranes was quantified by measuring the absorbance of haemoglobin (405 nm) released in the supernatant of each well. PBS and

0.01% (v/v) Triton X-100 were used as controls to establish 0% and 100% of haemolysis, respectively. Dose–response curves were fitted using nonlinear binding with the Hill slope equation and constraining the maximum to 100% using GraphPad Prism.

Inductively coupled plasma mass spectrometry (ICP-MS). For ICP-MS analysis, 0.2 mL 65% HNO_3 and 0.05 mL 30% H_2O_2 were added into a sample containing 1.1 mg of **penetratin-SNBD**. The sample was incubated at 95 °C for 30 min, diluted with deionized H_2O to a final volume of 5 mL and analyzed using an Agilent ICP-MS model 7800.

Protein labeling. Lyophilized mCCL2-Cys (100 µg) was dissolved in 20 mM Tris (pH 7.5) to a final concentration of 50 µM. Afterwards, **Pd-SNBD** in DMF was added to the mCCL2-Cys solution to reach a concentration of 200 µM (5 equiv.) in 5% DMF, and the sample was incubated at 37 °C. After 30 min, the reaction was quenched with 3-mercaptopropionic acid (800 µM) and the **mCCL2-SNBD** was purified using a Zeba™ spin desalting column (7K MWCO 0.5 mL). The labeling reaction was analyzed by mass spectrometry and SDS-PAGE electrophoresis in 4-12% NuPAGE Bis-Tris protein gels under non-reducing conditions. In-gel fluorescence was measured with a GelDoc™ BioRad imaging system using Alexa488 (for SNBD) and Cy5 (for the ladder) filters. Afterwards, gels were stained with Coomassie Blue G-250, de-stained in water, and imaged under white light.

2. Chemical Synthesis

Cys-benzodiazole amino acids and [(1,5-COD)Pd(CH_2TMS)$_2$]. Cys-benzodiazole amino acids were prepared according to the previously reported literature procedure.[1] [(1,5-COD)Pd(CH_2TMS)$_2$] was prepared according to the literature procedure.[2, 3]

Compound SNBD-Br. 4,7-Dibromo-2,1,3-benzothiadiazole (1.7 g, 5.88 mmol, 1 equiv.) were dissolved in HNO_3 conc. (30 ml) and heated to 90 °C for 5 h. Reaction mixture was then poured into ice/water (250 mL) and the resulting precipitate was collected by filtration. It was then purified by column chromatography (DCM:Hex 4:6 to 8:2) to afford **SNBD-Br** as a light-yellow powder (611 mg, 40%).

^{1}H NMR (500 MHz, $CDCl_3$) δ 8.50 (d, J = 8.1 Hz, 1H), 8.07 (d, J = 8.1 Hz, 1H).

^{13}C NMR (101 MHz, $CDCl_3$) δ 154.6, 145.8, 139.0, 130.3, 127.6, 123.0.

MS (ESI) for $C_6H_3BrN_3O_2S$; m/z $[M+H]^+$ calcd. 259.9; found 260.0.

Compound Pd-SNBD. In a 50 mL Schlenk flask equipped with a magnetic stir bar, RuPhos (60 mg, 0.13 mmol, 1.1 equiv.) and SNBD-Br (30 mg, 0.11 mmol, 1.0 equiv.) were dissolved in 2 mL dry THF. Solid (1,5-COD)Pd(CH_2SiMe_3)$_2$ (45 mg, 0.11 mmol, 1.0 equiv.) was dissolved in dry THF (1 mL) and added rapidly in one portion. The resulting solution was stirred for 16 h at r.t. After that, n-pentane (10 mL) was added, and the resulting mixture was transferred to a vial (20 mL) and placed into a –20 °C freezer for 3 h. The vial was then taken outside of the freezer and the resulting precipitate was collected, washed with pentane (3 × 5 mL), and dried under vacuum to afford **Pd-SNBD** as a yellow powder (66 mg, 72%).

Note: We stored our complexes at room temperature under nitrogen atmosphere which gave no diminished reactivity even after 3 months.

^{1}H NMR (400 MHz, CD_2Cl_2) δ 8.22 (d, *J* = 7.7 Hz, 1H), 7.71 (t, *J* = 8.4 Hz, 1H), 7.59 (t, *J* = 7.4 Hz, 1H), 7.49 (t, *J* = 7.3 Hz, 1H), 7.42 (d, *J* = 7.9 Hz, 2H), 6.88 (d, *J* = 6.9 Hz, 1H), 6.72 (d, *J* = 8.4 Hz, 1H), 6.61 (d, *J* = 8.4 Hz, 1H), 4.66 (dq, *J* = 18.2, 5.9 Hz, 2H), 2.38 – 2.26 (m, 1H), 2.12 – 1.96 (m, 2H), 1.89 – 1.64 (m, 7H), 1.60 (d, *J* = 5.7 Hz, 3H), 1.54 (s, 3H), 1.45 (d, *J* = 12.8 Hz, 2H), 1.37 (d, *J* = 5.8 Hz, 3H), 1.31 – 1.17 (m, 4H), 1.13 (d, *J* = 5.7 Hz, 4H), 1.04 (d, *J* = 13.2 Hz, 1H), 0.97 (d, *J* = 5.9 Hz, 3H), 0.92 – 0.85 (m, 1H).

^{13}C NMR (101 MHz, $CDCl_3$) δ 162.0 (s), 161.6 (s), 160.4 (s), 154.3 (s), 145.0 (d, *J* = 17.6 Hz), 144.3 (s), 136.7 (s), 136.3 (s), 133.9 (d, *J* = 3.7 Hz), 133.3 (s), 132.9 (s), 132.5 (d, *J* = 11.8 Hz), 131.3 (s), 130.6 (s), 126.8 (d, *J* = 5.6 Hz), 125.0 (s), 107.6 (s), 106.7 (s), 77.3 (s), 77.0 (s), 76.7 (s), 72.2 (s), 70.5 (s), 35.1 (d, *J* = 27.2 Hz), 33.3 (s), 33.0 (s), 29.7 (s), 27.6 (d, *J* = 48.1 Hz), 27.3–26.6 (m), 26.3 (s), 25.9 (dd, *J* = 36.0, 16.3 Hz), 22.5 (s), 22.0 (s), 21.6 (d, *J* = 15.0 Hz).

^{31}**P NMR** (162 MHz, $CDCl_3$) δ 36.8 (s), 35.2 (s).

HRMS (ESI) for $C_{36}H_{45}N_3O_4PSPd$; m/z: $[M-Br]^+$ calcd.: 752.1903, found: 752.1913.

Preparation of 2-chlorotrityl-hydrazino resin. The resin was prepared following the scheme below:

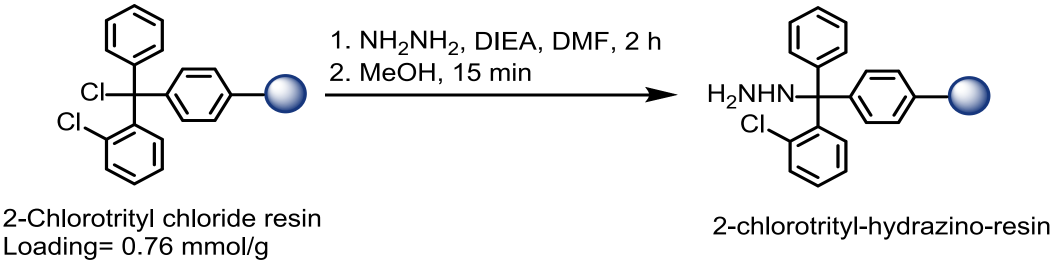

2-chlorotrityl chloride resin (132 mg, 0.76 mmol g^{-1}, 0.1 mmol) was swelled in DMF for 15 min and cooled to 0°C. A mixture of DIPEA (190 μL) and hydrazine hydrate (96 μL, 50%) in DMF (571 μL) was added slowly. Then, the suspension was stirred at r.t. and after 2 h, 115 μL MeOH were added and stirred for 15 min. Finally, the resin was washed with DMF, H_2O, DMF, MeOH, and Et_2O and dried under vacuum.

Synthesis of linear model peptide (P1). The synthesis of the linear peptide **P1** was carried out using SPPS on Rink Amide Protide resin (167 mg, loading 0.6 mmol g^{-1}, 0.1 mmol scale).[4] The resin was pre-swollen in DMF for 30 min and then transferred to the CSBio automated peptide synthesizer and coupled all amino acids with Fmoc-protected amino acids (10 equiv.) using HBTU and HOBt (both 10 equiv.) and 0.37 mL DIPEA. The couplings were carried out at 60 °C for 15 min. When the synthesis was completed, the peptide-bound resin was washed with DMF (5 mL × 3), MeOH (5 mL × 3), and DCM (5 mL × 3) and dried under vacuum. Subsequently, a mixture of TFA:H_2O:TIS (95:2.5:2.5, 7

mL for 0.05 mmol) was added to the resin, which was shaken for 3 h at r.t. The resin was filtered and washed with TFA (2 × 1 mL). To precipitate the peptide, the combined filtrates were added dropwise to cold Et_2O (25 mL for 0.05 mmol) followed by centrifugation at 4,000 rpm for 7 min. Then, Et_2O was decanted followed by dissolution of the peptide in ACN:H_2O (2.5:7.5) and lyophilized to isolate a white powder that was purified by RP-HPLC affording the product as a white powder (50 mg, 45%).

MS (ESI) for $C_{51}H_{82}N_{14}O_{12}S$; m/z $[M+2H]^{2+}$ calcd.: 558.7, found: 558.7.

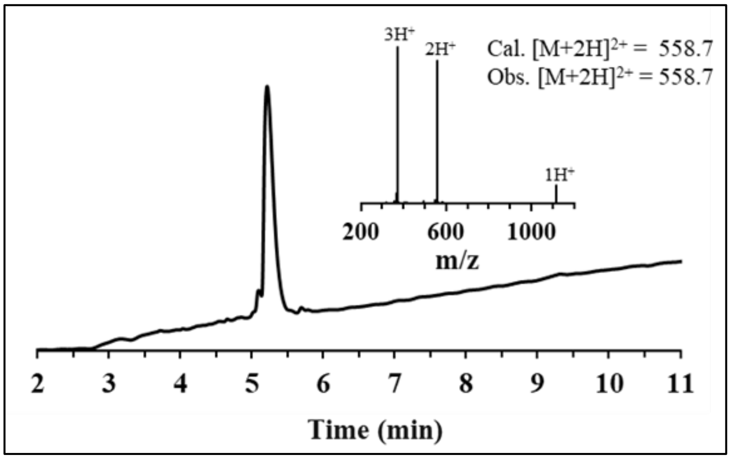

HPLC trace and MS analysis of pure **P1**.

Synthesis of P1-SNBD. To a 15 mL falcon tube was added **P1** (9.5 mL, 500 µM, 1.0 equiv.) as a solution in 3 M GdmCl ,100 mM Na_2HPO_4 buffer (pH 7.5) and **Pd-SNBD** (500 µL, 2.5 mM, 5 equiv.) as a solution in DMF. The reaction mixture was vortexed at 37 °C for 1 h. After ensuring the completion of the reaction, the whole reaction mixture was quenched with 3-MPA (10 equiv. compared to **Pd-SNBD**) and kept at 37 °C for 15 min. The reaction mixture was then spun down, and the crude mixture was purified in a Biotage® Selekt automated flash chromatography system to afford **P1-SNBD** as a yellow powder (2.9 mg, 48%).

MS (ESI) for $C_{57}H_{83}N_{17}O_{14}S_2$; m/z $[M+2H]^{2+}$ calcd.: 648.3, found: 648.2.

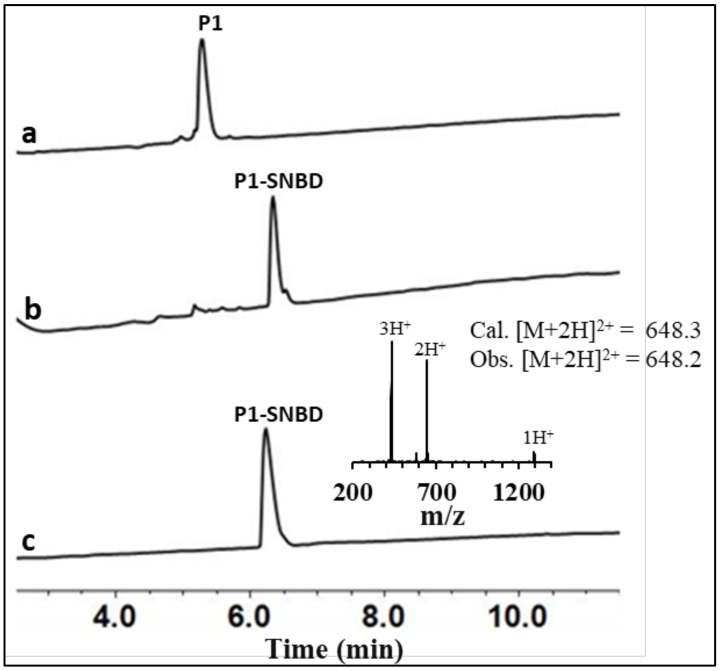

HPLC-MS analysis of crude **P1-SNBD** (b) and pure **P1-SNBD** (c).

Synthesis of the cyclic model peptide (cP1). First, the synthesis of the linear precursor was carried out in SPPS on hydrazide resin (132 mg, loading 0.76 mmol/g, 0.1 mmol scale). The resin was pre-swollen in DMF for 30 min and then transferred to the CSBio automated peptide synthesizer and coupled all amino acids with Fmoc-protected amino acids (10 equiv.) using HBTU and HOBt (both 10 equiv.) and 0.37 mL DIPEA. The couplings were carried out at 60 °C for 15 min. When the synthesis was completed, the peptide-bound resin was washed with DMF (5 mL × 3), MeOH (5 mL × 3), and DCM (5 mL × 3) and dried under vacuum. Subsequently, a mixture of TFA:H_2O:TIS (95:2.5:2.5, 7 mL for 0.05 mmol) was added to the resin, which was shaken for 3 h at r.t. The resin was filtered and washed with TFA (2 × 1 mL). To precipitate the peptide, the combined filtrates were added dropwise to cold Et_2O (25 mL for 0.05 mmol) followed by centrifugation at

4,000 rpm for 7 min. Then, Et_2O was decanted followed by dissolution of the peptide in ACN:H_2O (2.5:7.5) and lyophilized to isolate a white powder that was purified by RP-HPLC affording the non-cyclized product as a white powder (50 mg, 44%).

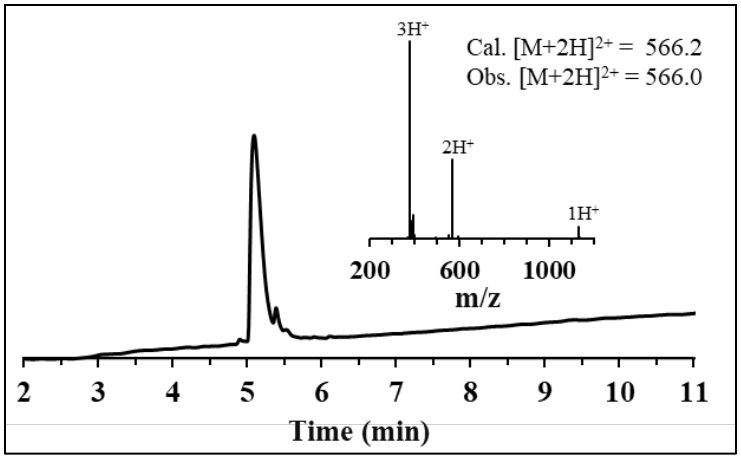

HPLC trace and MS analysis of linear precursor of **cP1**.

Non-cyclized **cP1** precursor (12 mg, 10.6 μmol) was dissolved in 6 M GdmCl, 0.2 M Na_2HPO_4 buffer (2 mL) at pH 3.0 and cooled down to -15 °C by placing in an ice/salt bath. 100 μL of $NaNO_2$ (10 equiv.) dissolved in water were added to the reaction mixture and allowed to react for 20 min at -15 °C with gentle mixing in repeated intervals. After 20 min 2 mL of MPAA (100 equiv.) in 6 M GdmCl, 0.2 M Na_2HPO_4 buffer (pH 7.2) was added to the mixture and gently mixed for 2 min. Finally, 1.2 mL of TCEP (50 equiv.) in 6 M GdmHCl, 0.2 M Na_2HPO_4 buffer (pH 7.2) was added. The pH of the reaction mixture was then adjusted to 7.2 using 1 N NaOH at 0 °C and then the mixture was kept at 25 °C and the reaction was monitored using HPLC-MS.[5] After 2 h ligation, the reaction mixture was diluted with H_2O and purified using a Biotage® Selekt automated flash chromatography system **cP1** as a white powder (7.6 mg, 65%).

MS (ESI) for $C_{51}H_{79}N_{13}O_{12}S$; m/z $[M+2H]^{2+}$ calcd.: 550.2, found: 550.1.

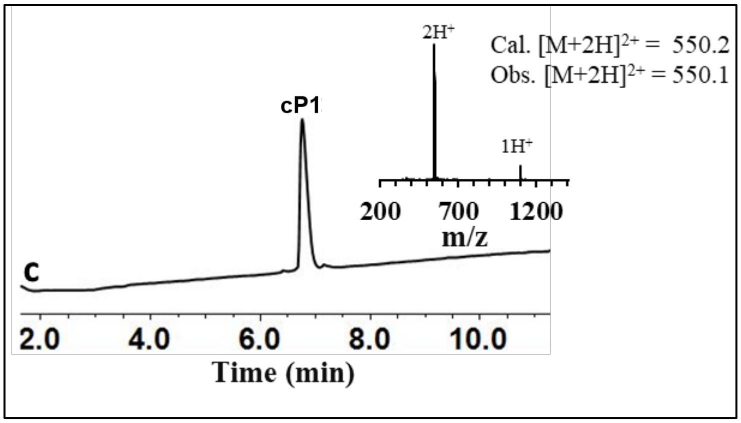

HPLC trace and MS analysis of pure **cP1**.

Synthesis of cP1-SNBD. To a 15 mL falcon tube was added **cP1** (8.5 mL, 750 μM, 1.0 equiv.) as a solution in 3 M GdmCl ,100 mM Na_2HPO_4 buffer (pH 7.5) and **Pd-SNBD** (450 μL, 3.75 mM, 5 equiv.) as a solution in DMF.[6] The reaction mixture was vortexed at 37 °C for 1 h. After ensuring the completion of the reaction, the whole reaction mixture was quenched with 3-MPA (5 equiv. compared to **Pd-SNBD**) and kept at 37 °C for 15 min. The reaction mixture was then spun down, and the crude mixture was purified in a Biotage® Selekt automated flash chromatography system to afford **cP1-SNBD** as a yellow powder (3.5 mg, 47%).

MS (ESI) for $C_{57}H_{80}N_{16}O_{14}S_2$; m/z $[M+H]^+$ calcd.: 1278.5, found: 1278.6; $[M+2H]^{2+}$ calcd.: 639.5, found: 639.5.

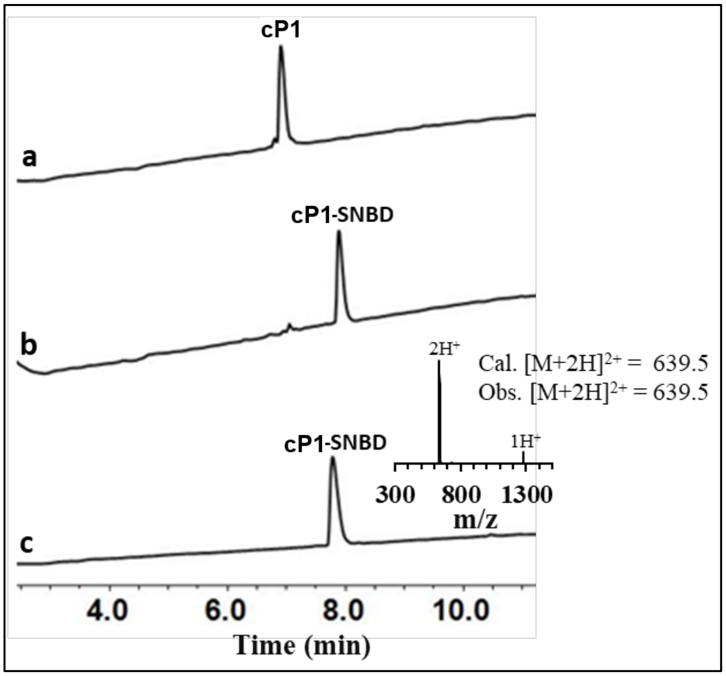

HPLC-MS analysis of crude **cP1-SNBD** (b) and pure **cP1-SNBD** (c).

Synthesis of Cys-free control peptide (tP1). The synthesis of the peptide **tP1** was carried out using SPPS on Rink Amide Protide resin (84 mg, loading 0.6 mmol g^{-1}, 0.1 mmol scale).[4] The resin was pre-swollen in DMF for 30 min and Fmoc-protected amino acids (5 equiv.) were coupled manually using HBTU/HOBt (5 equiv.) and 0.19 mL DIPEA. Couplings were carried out at 30 °C for 15 min. When the synthesis was completed, the peptide-bound resin was washed with DMF (5 mL × 3), MeOH (5 mL × 3), and DCM (5 mL × 3) and dried under vacuum. Subsequently, a mixture of TFA:H_2O:TIS (95:2.5:2.5, 7 mL for 0.05 mmol) was added to the resin, which was shaken for 3 h at r.t. The resin was filtered and washed with TFA (2 × 1 mL). To precipitate the peptide, the combined filtrates were added dropwise to cold Et_2O (25 mL for 0.05 mmol) followed by centrifugation at 4,000 rpm for 7 min. Then, Et_2O was decanted followed by dissolution of the peptide in

ACN:H_2O (2.5:7.5) and lyophilized to isolate a white powder that was purified by RP-HPLC affording the product as a white powder (26 mg, 48%).

MS (ESI) for $C_{51}H_{82}N_{14}O_{12}S$; m/z $[M+2H]^{2+}$ calcd.: 542.7, found: 542.7.

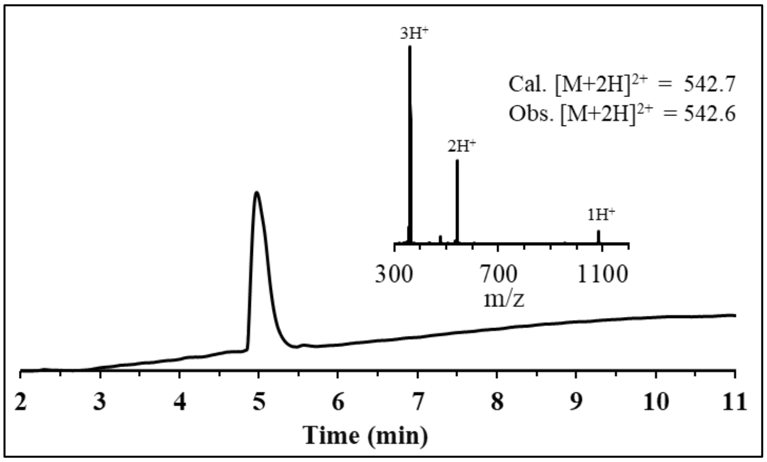

HPLC trace and MS analysis of pure **tP1**.

Synthesis of unlabeled TAT. The synthesis of TAT was carried out using SPPS on Rink Amide Protide resin (167 mg, loading 0.6 mmol g^{-1}, 0.1 mmol scale). The resin was swollen in DMF for 30 min and then removed the Fmoc group using 20% piperidine in DMF (3 cycles) and coupled the Fmoc-L-Gln(Trt)-OH (10 equiv.), using HBTU and HOBt (both 10 equiv.) and 0.37 mL DIPEA. The coupling was carried out for 20 min. The process was repeated for the remaining amino AAs in the sequence. When the synthesis was completed, the peptide-bound resin was washed with DMF (5 mL × 3), MeOH (5 mL × 3), and DCM (5 mL × 3) and dried under vacuum. Subsequently, a mixture of TFA:H_2O:TIS (95:2.5:2.5, 7 mL for 0.05 mmol) was added to the resin, which was shaken for 3 h at r.t. The resin was filtered and washed with TFA (2 × 1 mL). To precipitate the peptide, the combined filtrates were added dropwise to cold Et_2O (25 mL for 0.05 mmol) followed by centrifugation at 4,000 rpm for 7 min. Then, Et_2O was decanted followed by

dissolution of the peptide in ACN:H_2O (2.5:7.5) and lyophilized to isolate a white powder that was purified by RP-HPLC affording the non-cyclized product as a white powder (71 mg, 40%).

HRMS (ESI) for $C_{74}H_{134}N_{36}O_{16}$. m/z $[M+2H]^{2+}$ calcd.: 892.5389, found: 892.5501; $[M+3H]^{3+}$ calcd.: 595.3593, found: 595.3683; $[M+4H]^{4+}$ calcd.: 446.7694, found: 446.7775.

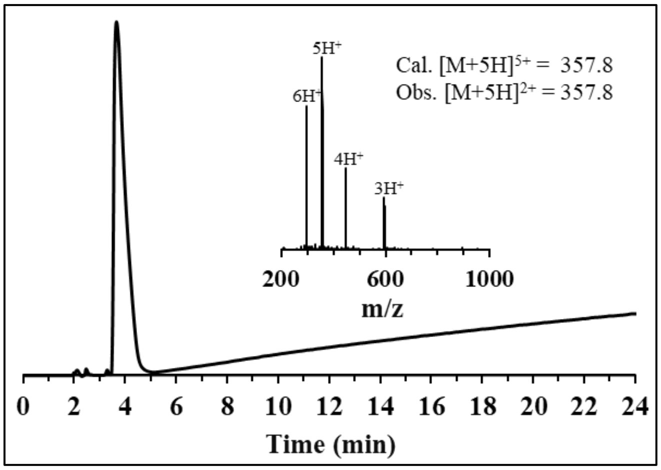

HPLC trace and MS analysis of pure unlabeled TAT.

Synthesis of unlabeled penetratin. The synthesis of penetratin was carried out using SPPS on Rink Amide Protide resin (167 mg, loading 0.6 mmol g^{-1}, 0.1 mmol scale). The resin was swollen in DMF for 30 min and then removed the Fmoc group using 20% piperidine in DMF (3 cycles) and coupled the Fmoc-L-Lys(Boc)-OH (10 equiv.), using HBTU and HOBt (both 10 equiv.) and 0.37 mL DIPEA. The coupling was carried out for 20 min. The process was repeated for the remaining amino AAs in the sequence. When the synthesis was completed, the peptide-bound resin was washed with DMF (5 mL × 3), MeOH (5 mL × 3), and DCM (5 mL × 3) and dried under vacuum. Subsequently, a mixture of TFA:H_2O:TIS (95:2.5:2.5, 7 mL for 0.05 mmol) was added to the resin, which was

shaken for 3 h at r.t. The resin was filtered and washed with TFA (2 × 1 mL). To precipitate the peptide, the combined filtrates were added dropwise to cold Et_2O (25 mL for 0.05 mmol) followed by centrifugation at 4,000 rpm for 7 min. Then, Et_2O was decanted followed by dissolution of the peptide in ACN:H_2O (2.5:7.5) and lyophilized to isolate a white powder that was purified by RP-HPLC affording the non-cyclized product as a white powder (148 mg, 66%).

HRMS (ESI) for $C_{104}H_{169}N_{35}O_{19}S$. m/z $[M+2H]^{2+}$ calcd.: 1123.1527, found: 1123.6608; $[M+3H]^{3+}$ calcd.: 749.1018, found: 749.4424; $[M+4H]^{4+}$ calcd.: 562.0764, found: 562.3348.

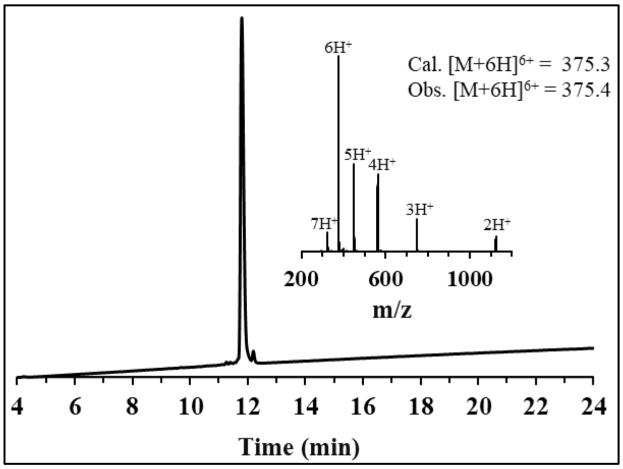

HPLC trace and MS analysis of pure unlabeled penetratin.

Synthesis of unlabeled peptide sC18. The synthesis of sC18 was carried out using SPPS on Rink Amide Protide resin (167 mg, loading 0.6 mmol g^{-1}, 0.1 mmol scale). The resin was swollen in DMF for 30 min and then removed the Fmoc group using 20% piperidine in DMF (3 cycles) and coupled the Fmoc-L-Lys(Boc)-OH (10 equiv.), using HBTU and HOBt (both 10 equiv.) and 0.37 mL DIPEA. The coupling was carried out for 20 min. The process was repeated for the remaining amino AAs in the sequence. When

the synthesis was completed, the peptide-bound resin was washed with DMF (5 mL × 3), MeOH (5 mL × 3), and DCM (5 mL × 3) and dried under vacuum. Subsequently, a mixture of TFA:H_2O:TIS (95:2.5:2.5, 7 mL for 0.05 mmol) was added to the resin, which was shaken for 3 h at r.t. The resin was filtered and washed with TFA (2 × 1 mL). To precipitate the peptide, the combined filtrates were added dropwise to cold Et_2O (25 mL for 0.05 mmol) followed by centrifugation at 4,000 rpm for 7 min. Then, Et_2O was decanted followed by dissolution of the peptide in ACN:H_2O (2.5:7.5) and lyophilized to isolate a white powder that was purified by RP-HPLC affording the non-cyclized product as a white powder (110 mg, 53%).

HRMS (ESI) for $C_{92}H_{169}N_{35}O_{19}$ m/z $[M+2H]^{2+}$ calcd.: 1035.1667, found: 1035.6735; $[M+3H]^{3+}$ calcd.: 690.4445 found: 690.7853; $[M+4H]^{4+}$ calcd.: 518.0834, found: 518.3416.

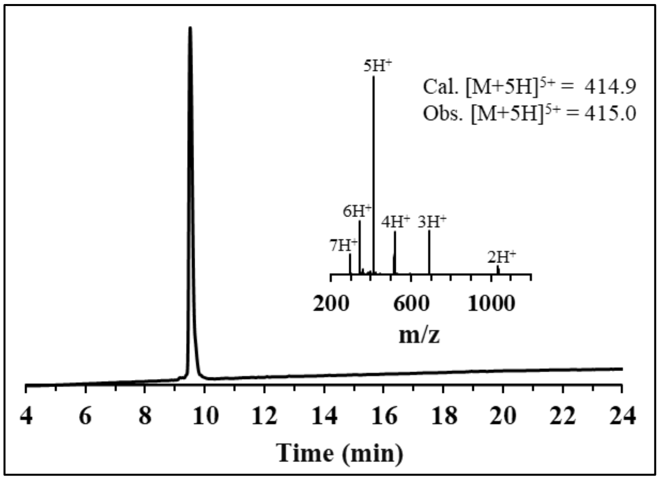

HPLC trace and MS analysis of pure unlabeled sC18.

Synthesis of TAT-SNBD. The synthesis of **TAT-SNBD** was carried out using SPPS on Rink Amide Protide resin (167 mg, loading 0.6 mmol g^{-1}, 0.1 mmol scale) using Fmoc-L-Cys(Trt)-OH as the C-terminal amino acid and the same conditions as detailed above.

The crude peptide powder was purified by RP-HPLC affording the product as a white powder (94 mg, 50%).

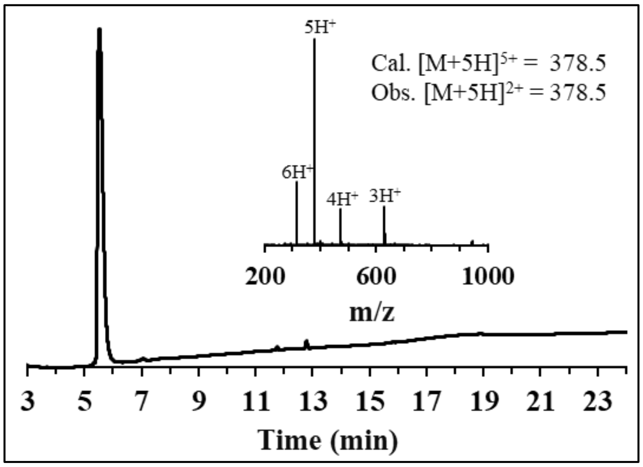

HPLC trace and MS analysis of pure unlabeled TAT-Cys.

To a 50 mL falcon tube was added TAT-Cys (15 mL, 250 μM, 1.0 equiv.) as a solution in 20 mM Tris buffer (pH 7.5) and **Pd-SNBD** (1.7 mL, 1.25 mM, 5 equiv.) as a solution in DMF (total 10% DMF). The reaction mixture was vortexed and kept at 37 °C for 1 h. The progress of reaction was monitored using analytical HPLC-MS. After completion of the reaction, the reaction mixture was lyophilized and dissolved in 3 mL 6 M GdmCl, 200 mM Na_2HPO_4 buffer (pH 7.2) and then quenched with 3-MPA (10 equiv. compared to **Pd-SNBD**), kept at 37 °C for 15 min. After the incubation, the reaction mixture was centrifuged down and the crude was purified using RP-HPLC to afford **TAT-SNBD** as a yellow powder (2.4 mg, 30%).

HRMS (ESI) for $C_{83}H_{140}N_{40}O_{19}S_2$. m/z $[M+3H]^{3+}$ calcd.: 689.3553, found: 689.6968; $[M+4H]^{4+}$ calcd.: 517.2665, found: 517.5251.

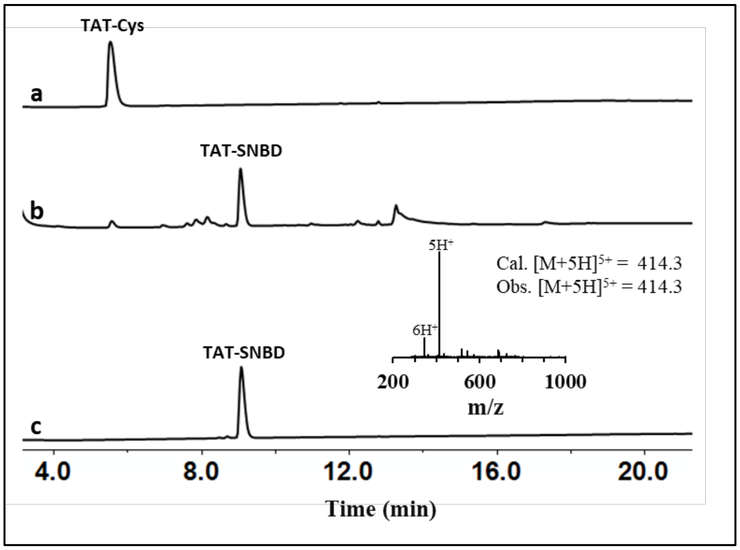

HPLC-MS analysis of TAT-Cys (a), crude **TAT-SNBD** (b) and pure **TAT-SNBD** (c).

Synthesis of penetratin-SNBD. The synthesis of **penetratin-SNBD** was carried out using SPPS on Rink Amide Protide resin (167 mg, loading 0.6 mmol g^{-1}, 0.1 mmol scale) using Fmoc-L-Cys(Trt)-OH as the C-terminal amino acid and the same conditions as detailed above. The crude peptide was purified by RP-HPLC affording the product as a white powder (122 mg, 52%).

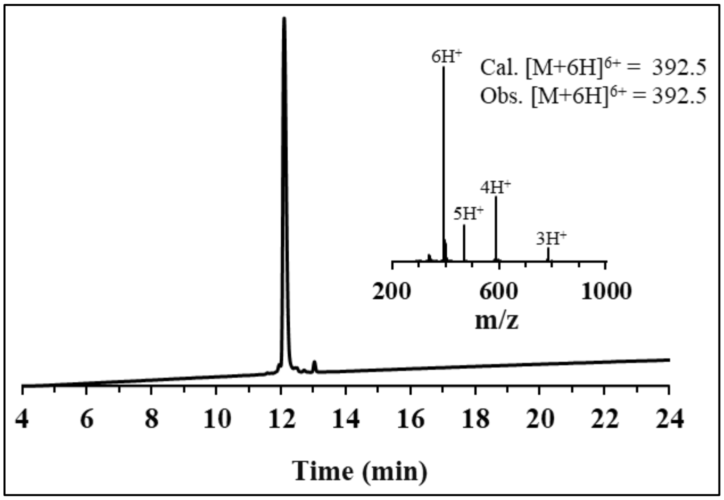

HPLC trace and MS analysis of pure unlabeled penetratin-Cys.

To a 50 mL falcon tube was added penetratin-Cys (12 mL, 250 μM, 1.0 equiv.) as a solution in 20 mM Tris buffer (pH 7.5) and **Pd-SNBD** (1.36 mL, 1.25 mM, 5 equiv.) as a solution in DMF (total 10% DMF). The reaction mixture was vortexed and kept at 37 °C for 1 h. The progress of reaction was monitored using analytical HPLC-MS. After completion of the reaction, the crude mixture was lyophilized and dissolved in 3 mL of 6 M GdmCl, 200 mM Na_2HPO_4 buffer (pH 7.2) and quenched with 3-MPA (10 equiv. compared to **Pd-SNBD**) at 37 °C for 15 min. The mixture was centrifuged down and the crude was purified using RP-HPLC to afford **penetratin-SNBD** as a yellow powder (2.8 mg, 35%).

HRMS (ESI) for $C_{113}H_{175}N_{39}O_{22}S_3$ m/z $[M+2H]^{2+}$ calcd.: 1264.1468, found: 1264.6582; $[M+3H]^{3+}$ calcd.: 843.0979, found: 843.4382; $[M+4H]^{4+}$ calcd.: 632.5738, found: 632.8313.

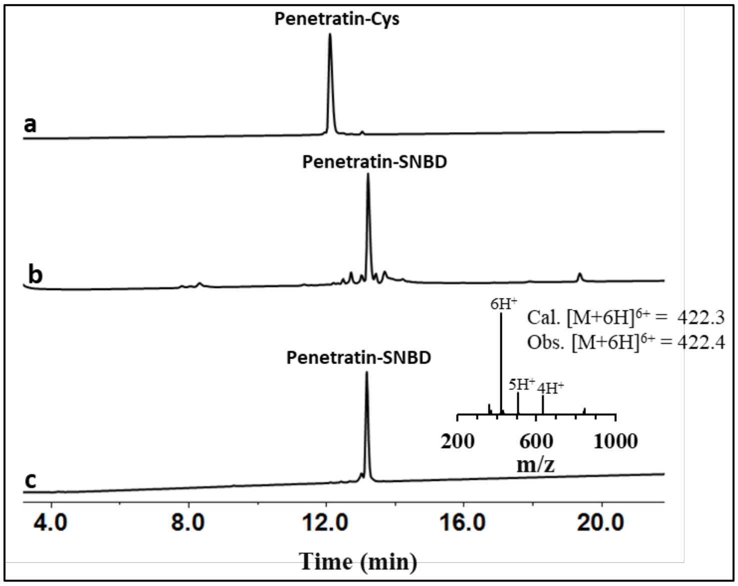

HPLC-MS analysis of penetratin-Cys (a), crude **penetratin-SNBD** (b) and pure **penetratin-SNBD** (c).

Synthesis of sC18-SNBD. The synthesis of **sC18-SNBD** was carried out using SPPS on Rink Amide Protide resin (167 mg, loading 0.6 mmol g^{-1}, 0.1 mmol scale) using Fmoc-L-Cys(Trt)-OH as the C-terminal amino acid and the same conditions as detailed above. The crude peptide powder was purified by RP-HPLC affording the product as a white powder (104 mg, 48%).

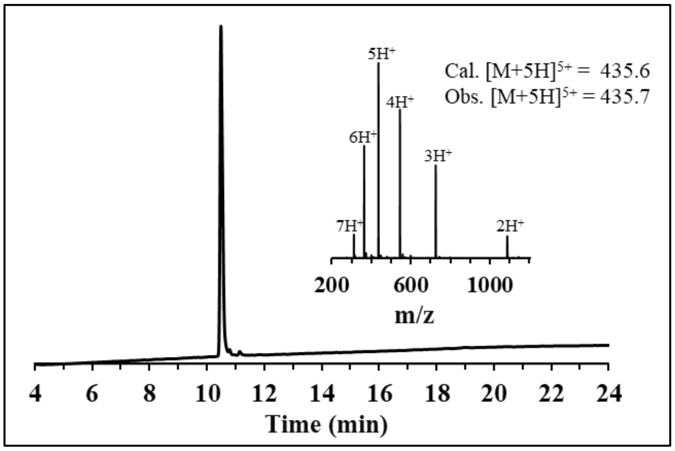

HPLC trace and MS analysis of pure unlabeled sC18-Cys.

To a 50 mL falcon tube was added sC18-Cys (13.5 mL, 250 μM, 1.0 equiv.) as a solution in 20 mM Tris buffer (pH 7.5) and **Pd-SNBD** (1.5 mL, 1.25 mM, 5 equiv.) as a solution in DMF (total 10% DMF). The reaction mixture was vortexed and kept at 37 °C for 1 h. The progress of reaction was monitored using analytical HPLC-MS. After completion of the reaction, the reaction mixture was lyophilized and dissolved in 3 mL 6 M GdmCl, 200 mM Na_2HPO_4 buffer (pH 7.2) and then quenched with 3-MPA (10 equiv. compared to **Pd-SNBD**), kept at 37 °C for 15 min. After the incubation, the reaction mixture was centrifuged down and the decant was directly purified using RP-HPLC to afford **sC18-SNBD** as a yellow powder (2.7 mg, 33%).

HRMS (ESI) for $C_{101}H_{175}N_{39}O_{22}S_2$ m/z $[M+2H]^{2+}$ calcd.: 1176.1608 found: 1176.6690; $[M+3H]^{3+}$ calcd.: 784.4405 found: 784.7810; $[M+4H]^{4+}$ calcd.: 588.5804, found: 588.8397.

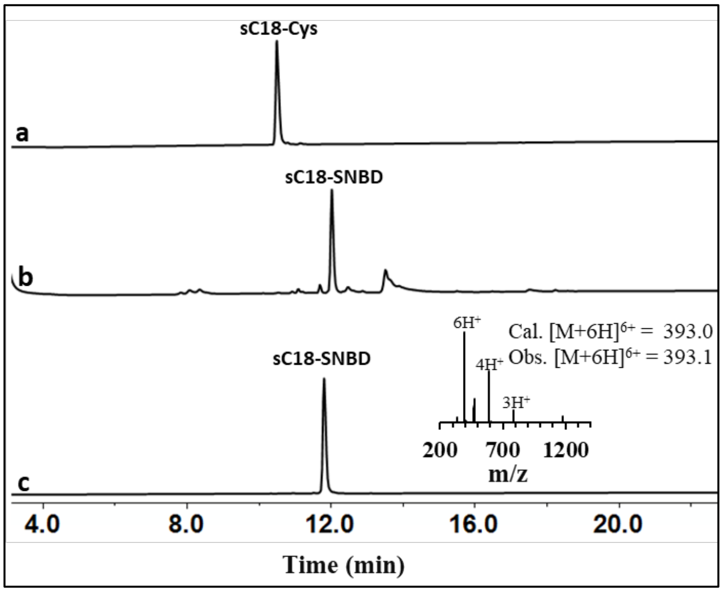

HPLC-MS analysis of sC18-Cys (a), crude **sC18-SNBD** (b) and pure **sC18-SNBD** (c).

3. Supplementary Figures

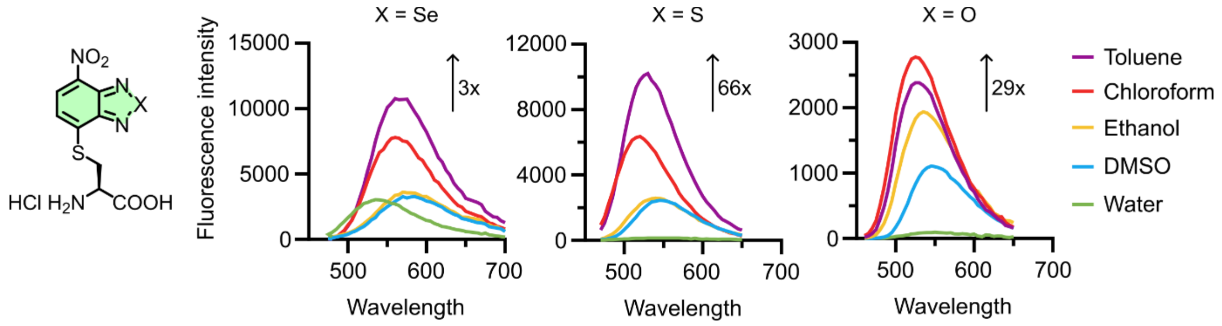

Figure S1. Representative fluorescence emission spectra of Cys-benzodiazoles. Spectra of Cys-based amino acids bearing benzo-2,1,3-selenadiazole, benzo-2,1,3-thiadiazole and benzo-2,1,3-oxadiazole cores (10 μM) were recorded in toluene (purple), chloroform (red), ethanol (yellow), DMSO (blue), and water (green) after excitation at 460 nm. Fluorescence-fold increase ratios between hydrophobic and hydrophilic environments were determined by relating their emission intensities in toluene and water.

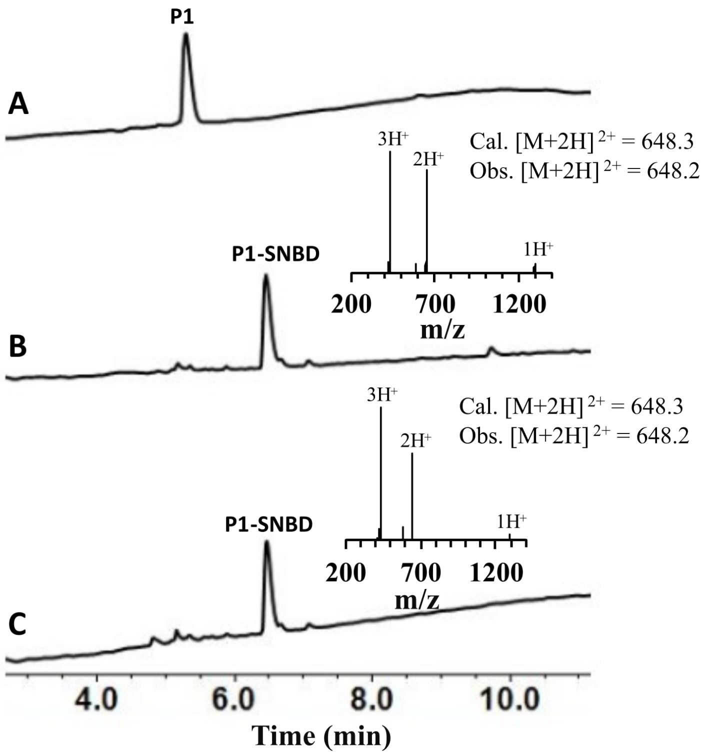

Figure S2. HPLC-MS analysis of peptide P1 labeling using fresh Pd-SNBD and Pd-SNBD after 3 months of storage. A) HPLC trace of peptide **P1**. B) Crude reaction after 1 h using fresh **Pd-SNBD** (5 equiv.), 5% DMF, 6 M GdmCl 0.2 mM Na_2HPO_4 buffer, pH 7.5, 37 °C (> 90% conversion). C) Crude reaction after 1 h using stored **Pd-SNBD** (5 equiv.), 5% DMF, 6 M GdmCl 0.2 mM Na_2HPO_4 buffer, pH 7.5, 37 °C (> 90% conversion). UV detection: 220 nm.

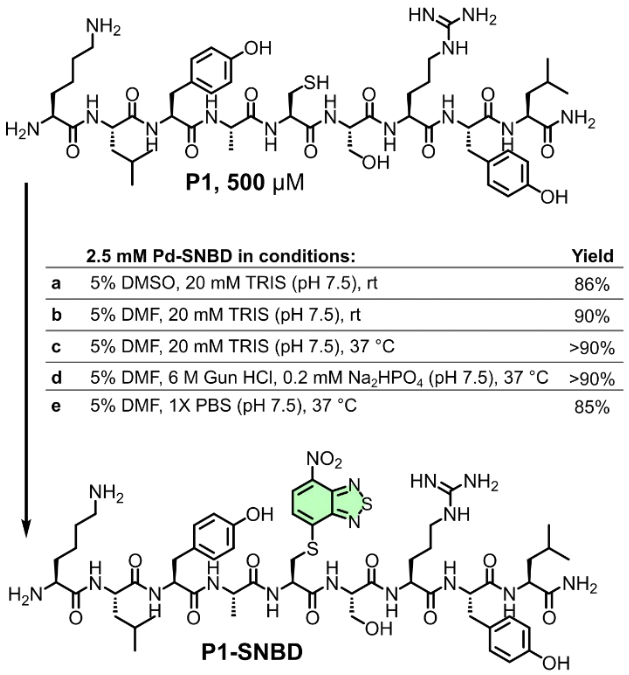

	2.5 mM Pd-SNBD in conditions:	Yield
**a**	5% DMSO, 20 mM TRIS (pH 7.5), rt	86%
**b**	5% DMF, 20 mM TRIS (pH 7.5), rt	90%
**c**	5% DMF, 20 mM TRIS (pH 7.5), 37 °C	>90%
**d**	5% DMF, 6 M Gun HCl, 0.2 mM Na_2HPO_4 (pH 7.5), 37 °C	>90%
**e**	5% DMF, 1X PBS (pH 7.5), 37 °C	85%

Figure S3. Optimization of the reaction conditions for labeling of unprotected peptides with Pd-SNBD. In 1.5 mL tubes, **P1** (10 μL, 2 mM, 1.0 equiv.) was dissolved in the buffer indicated in the Table and **Pd-SNBD** (5 μL, 20 mM, 5 equiv.). The final concentrations of the main components were: **P1** (0.2 mM); **Pd-SNBD** (1 mM). The reaction mixtures were vortexed and kept at r.t. or 37 °C for 1 h. Next, the reaction was treated with 3-MPA (10 equiv. compared to **Pd-SNBD**) and kept at 37 °C for 5 min. The yields are based on the integration of the crude reaction from HPLC-MS analysis.

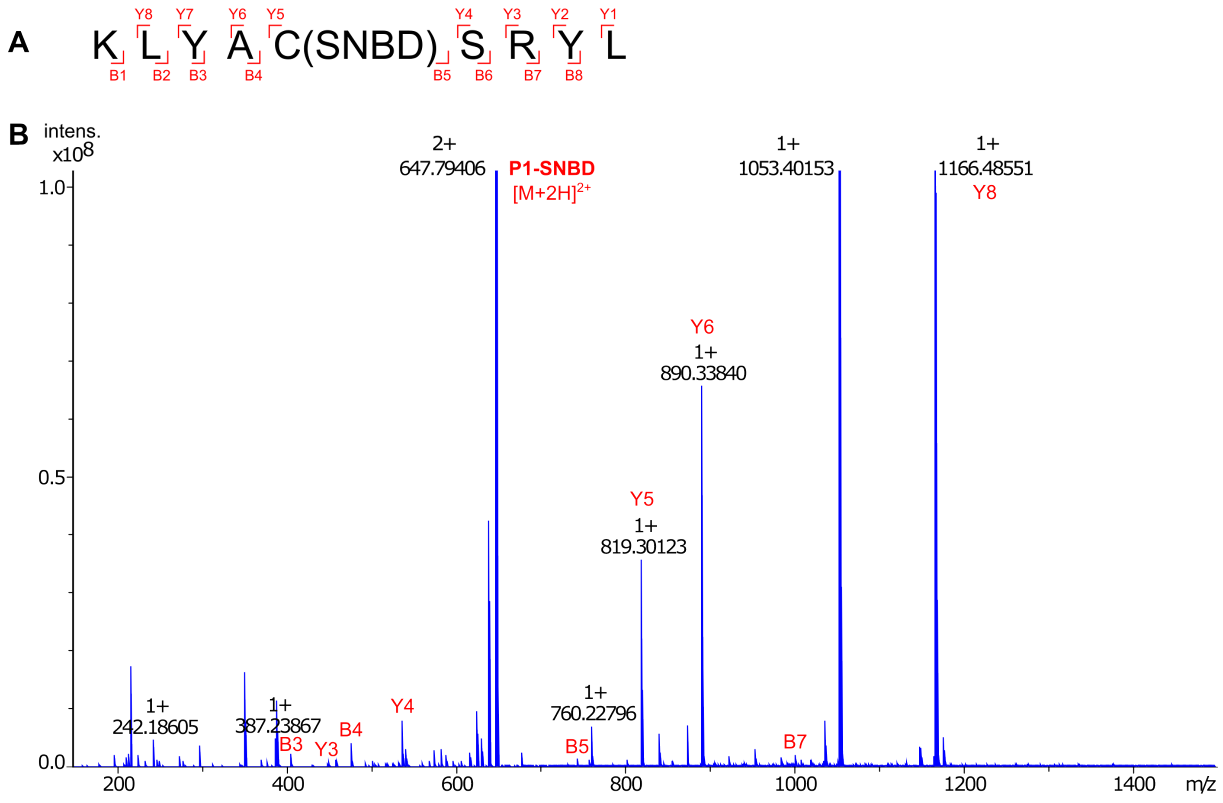

C

Name	Ion Type	Theoretical Mass	Observed Mass	Mass Difference (Da)	Mass Difference (ppm)
B3	B	404.242350	404.24191	-0.000436	-1.079715
B4	B	475.279460	475.27897	-0.000486	-1.023538
B5	B	757.267597	757.26645	-0.001143	-1.509990
B7	B	1000.400737	1000.39869	-0.002043	-2.042648
B8	B	1163.464067	1163.46323	-0.000833	-0.716366
Y3	Y	449.275049	449.27453	-0.000515	-1.147330
Y4	Y	536.307079	536.30649	-0.000585	-1.091663
Y5	Y	818.295216	818.29395	-0.001262	-1.542801
Y6	Y	889.332326	889.33112	-0.001202	-1.352100
Y8	Y	1165.479716	1165.47820	-0.001512	-1.297720

Figure S4. MS/MS analysis of P1-SNBD. A) Sequence of **P1-SNBD** with fragments Y1-Y8 (top) and B1-B8 (bottom) indicated in red. B) Full MS/MS spectrum of **P1-SNBD**, with identified fragments annotated in red. C) Theoretical and observed masses of detected ions, with the mass difference in Da and ppm.

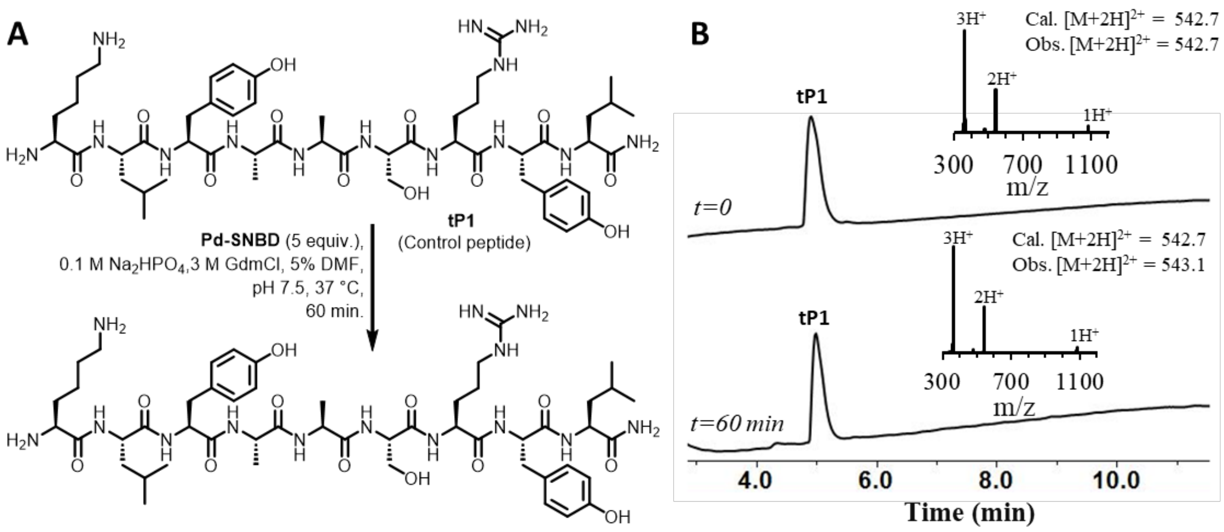

Figure S5. Incubation of control peptide tP1 under optimized labeling conditions. A) Synthetic scheme including labeling conditions for control peptide **tP1**. B) HPLC traces of **tP1** (top) and the resulting product (bottom) after reaction with Pd-SNBD. UV detection: 220 nm.

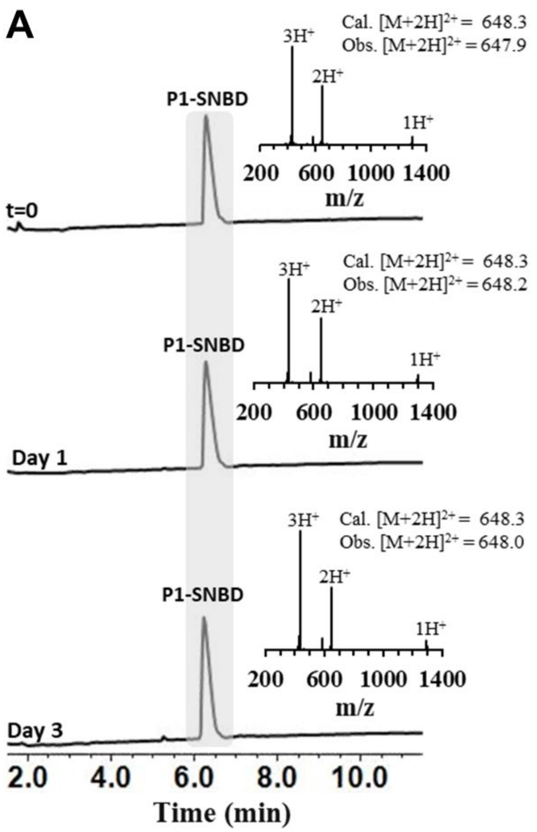

Figure S6. HPLC-MS stability analysis of pure P1-SNBD and cP1-SNBD in PBS at r.t. for 3 days. A) Stability analysis of **P1-SNBD**. B) Stability analysis of **cP1-SNBD**. UV detection: 220 nm.

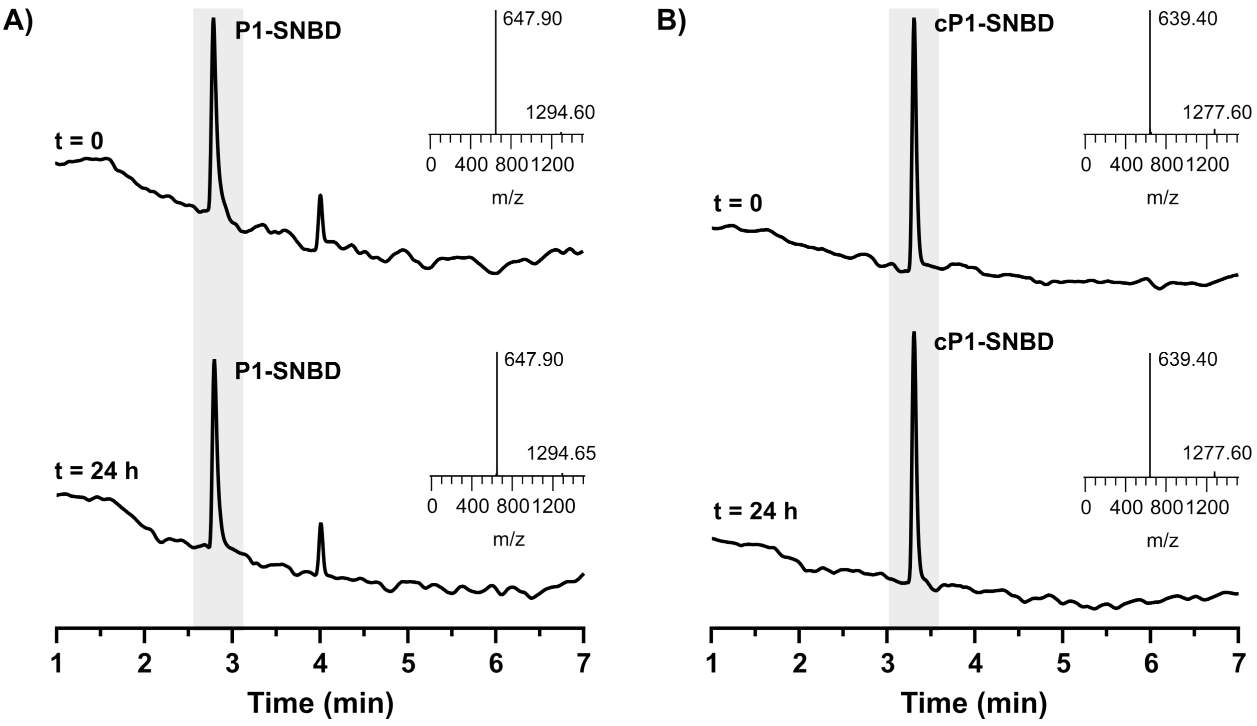

Figure S7. HPLC-MS stability analysis of pure P1-SNBD and cP1-SNBD in cell medium at 37°C for 24 h. A) Stability analysis of **P1-SNBD**. B) Stability analysis of **cP1-SNBD**.

	3 h	24 h	48 h
5 mM glutathione	>95%	>95%	>95%
1 mM NADH	>95%	>95%	>95%

Figure S8. Stability analysis of P1-SNBD under physiological conditions. The peptide **P1SNBD** (250 μM) was incubated in PBS (pH 7.8) at 37°C for the indicated timepoints and in the presence of the indicated bioanalytes. Peptide stability was determined by HPLC and quantified as a percentage relative to **P1-SNBD** in the absence of bioanalytes.

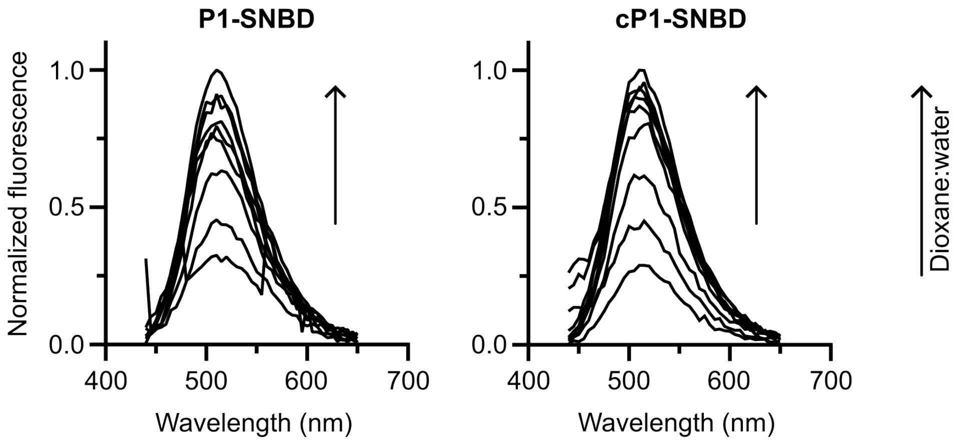

Figure S9. Representative emission spectra of fluorogenic peptides P1-SNBD and cP1-SNBD. Normalized fluorescence spectra of **P1-SNBD** (left, 10 µM) and **cP1-SNBD** (right, 10 µM) were recorded in mixtures of dioxane:water. Excitation wavelength: 460 nm.

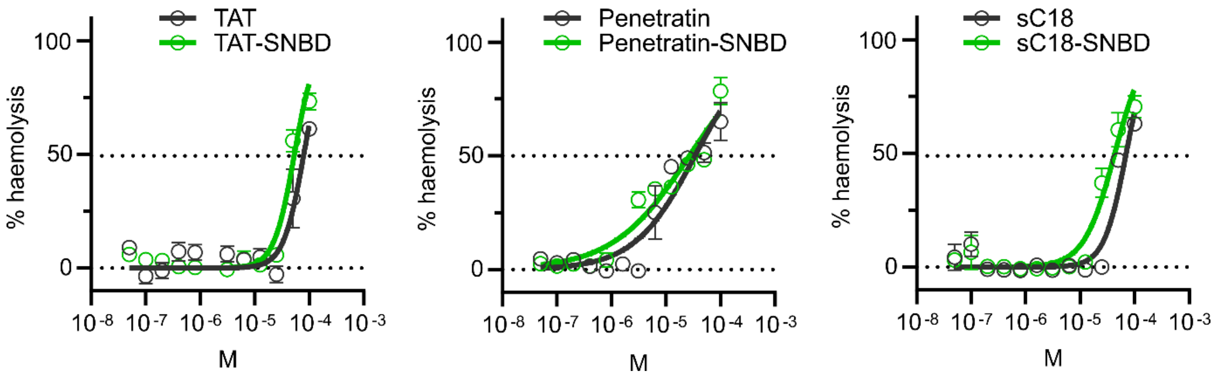

Figure S10. Haemolytic activity of unlabeled and SNBD-labeled CPPs. Human red blood cells in suspension (0.5% v/v in PBS) were treated with serially diluted peptides for 1 h at 37 °C. The disruption of red blood cell membranes was quantified by measuring the absorbance of haemoglobin (405 nm) released in the medium. PBS and 0.01% (v/v) Triton X-100 were used as controls to establish 0% and 100% of haemolysis, respectively. Data are presented are means ± SEM and dose–response curves are fitted using nonlinear binding with the Hill slope equation and constraining the maximum to 100% using GraphPad Prism.

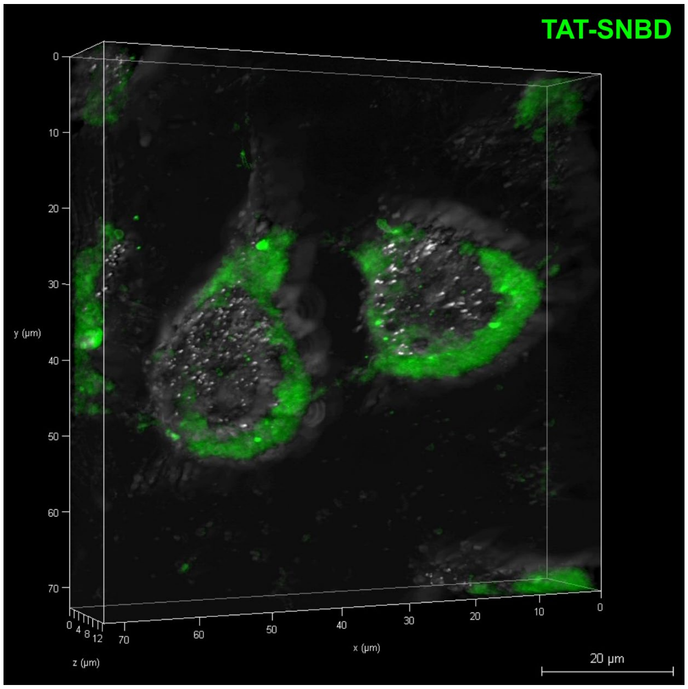

Figure S11. Z-stack imaging of HeLa cells treated with TAT-SNBD. Representative fluorescence microscopy 3D reconstructions of HeLa cells after treatment with **TAT-SNBD** (10 µM) and imaged by confocal microscopy (405/515 nm) after incubation at 37 °C with 5% CO_2 for 30 min. Stack images were acquired across 15.91 µm. Scale bars: 20 µm.

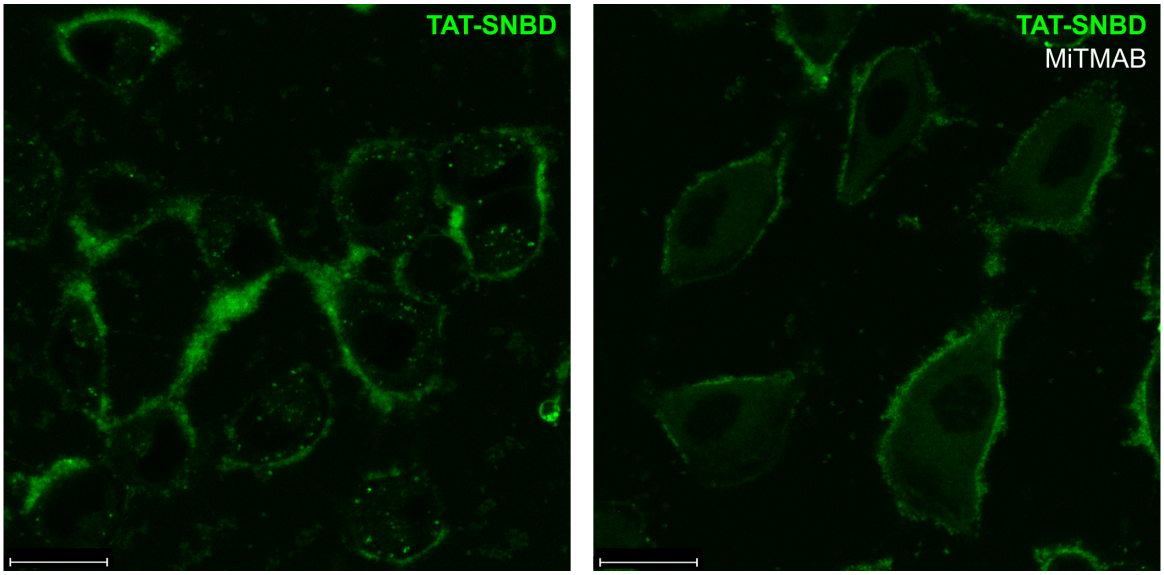

Figure S12. Imaging of HeLa cells after treatment with TAT-SNBD and inhibition of endocytosis. Representative fluorescence confocal microscopy images of HeLa cells after treatment with **TAT-SNBD** (10 μM) with or without the endocytosis inhibitor MiTMAB^{TM} (15 μM). Images were acquired after incubation at 37 °C with 5% CO_2 for 30 min (exc/em: 405/515 nm). Scale bars: 25 μm.

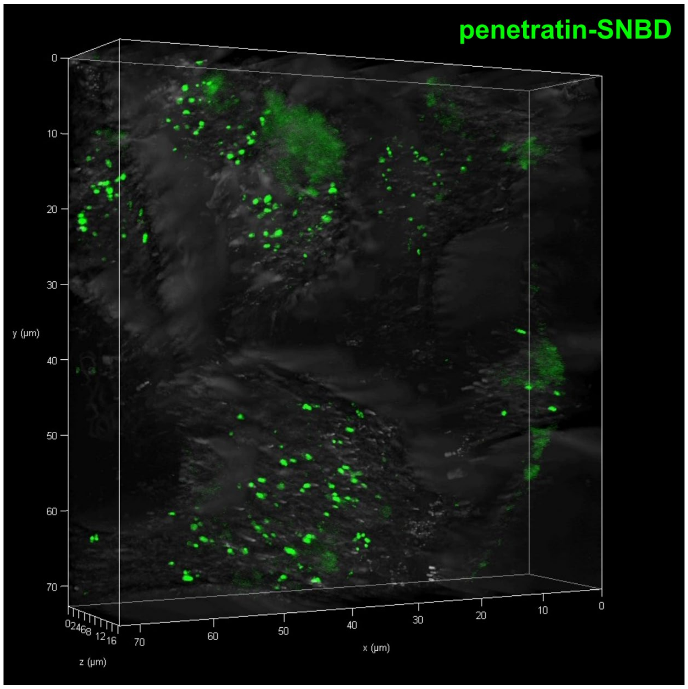

Figure S13. Z-stack imaging of HeLa cells treated with penetratin-SNBD. Representative fluorescence microscopy 3D reconstructions of HeLa cells after treatment with **penetratin-SNBD** (10 µM) and imaged by confocal microscopy (405/515 nm) after incubation at 37 °C with 5% CO_2 for 30 min. Stack images were acquired across 18.01 µm. Scale bars: 20 µm.

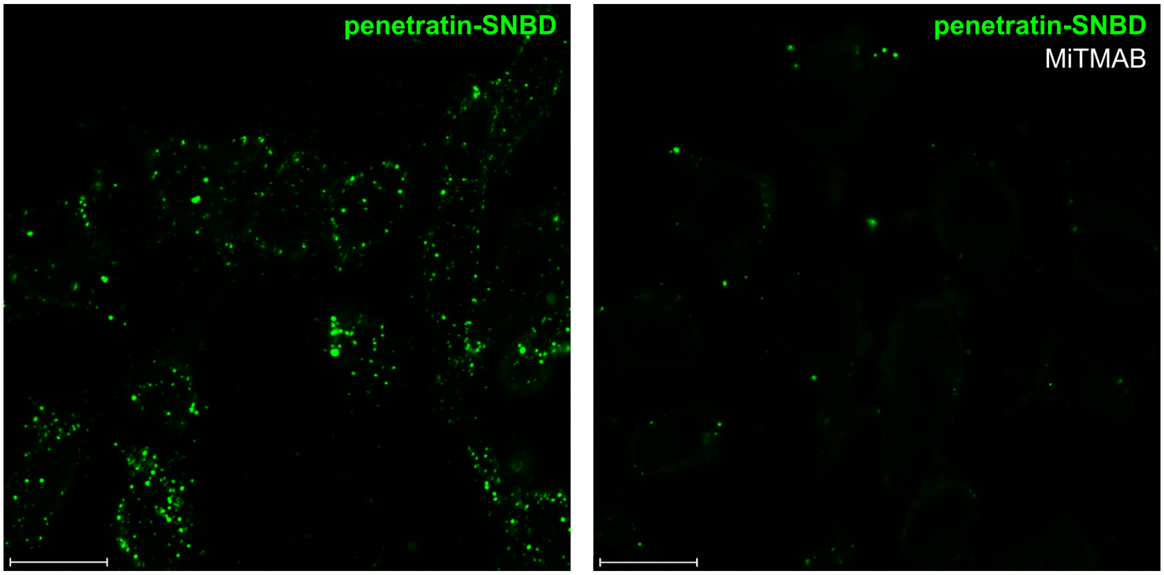

Figure S14. Imaging of HeLa cells after treatment with penetratin-SNBD and inhibition of endocytosis. Representative fluorescence confocal microscopy images of HeLa cells after treatment with **penetratin-SNBD** (10 μM) with or without the endocytosis inhibitor MiTMAB™ (15 μM). Images were acquired after incubation at 37 °C with 5% CO_2 for 30 min (exc/em: 405/515 nm). Scale bars: 25 μm.

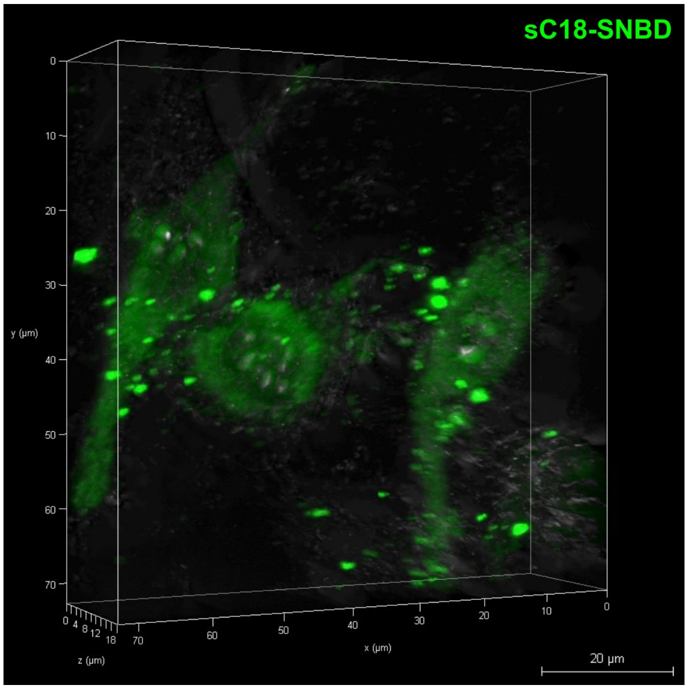

Figure S15. Z-stack imaging of HeLa cells treated with sC18-SNBD. Representative fluorescence microscopy 3D reconstructions of HeLa cells after treatment with **sC18-SNBD** (10 μM) and imaged by confocal microscopy (405/515 nm) after incubation at 37 °C with 5% CO_2 for 30 min. Stack images were acquired across 18.91 μm. Scale bars: 20 μm.

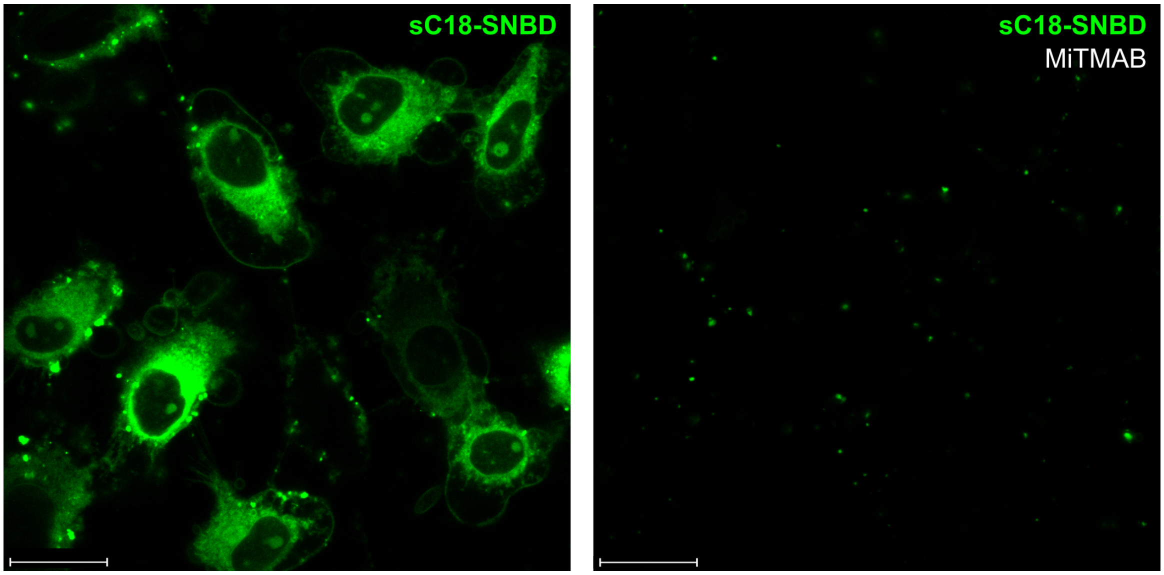

Figure S16. Imaging of HeLa cells after treatment with sC18-SNBD and inhibition of endocytosis. Representative fluorescence confocal microscopy images of HeLa cells after treatment with **sC18-SNBD** (10 µM) with or without the endocytosis inhibitor MiTMAB™ (15 µM). Images were acquired after incubation at 37 °C with 5% CO_2 for 30 min (exc/em: 405/515 nm). Scale bars: 25 µm.

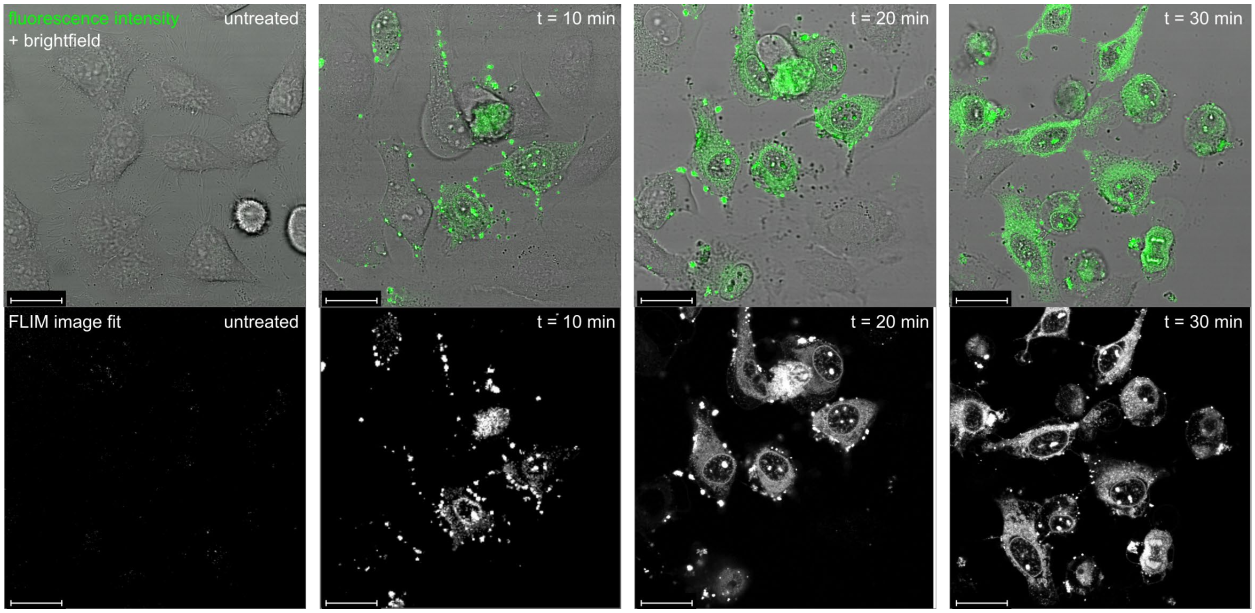

Figure S17. Fluorescence intensity thresholds for FLIM imaging and analysis. Merged brightfield and fluorescence images (top panels) and FLIM images (bottom panels) of HeLa cells after incubation or not with **sC18-SNBD** (10 µM) and acquisition by confocal microscopy (exc/em: 440/515). A low threshold of 20 counts was established for the FLIM fitted images. Scale bars: 25 µm.

4. Movie Legends

Movie S1. Time-lapse fluorescence imaging of TAT-SNBD. Time-lapse fluorescence confocal microscopy images of HeLa cells after incubation with **TAT-SNBD** (10 μM, green) and the lysosomal counterstain LysoTracker (red). **TAT-SNBD** was added, and fluorescence images were immediately recorded. Excitation lasers: 405 nm (for **TAT-SNBD**), 561 nm (for LysoTracker Red). Movie recorded for 20 min and compressed (jpeg) at 10 fps.

Movie S2. Time-lapse fluorescence imaging of penetratin-SNBD. Time-lapse fluorescence confocal microscopy images of HeLa cells after incubation with **penetratin-SNBD** (10 μM, green) and the lysosomal counterstain LysoTracker (red). **Penetratin-SNBD** was added, and fluorescence images were immediately recorded. Excitation lasers: 405 nm (for **penetratin-SNBD**), 561 nm (for LysoTracker Red). Movie recorded for 20 min and compressed (jpeg) at 10 fps.

Movie S3. Time-lapse fluorescence imaging of sC18-SNBD. Time-lapse fluorescence confocal microscopy images of HeLa cells after incubation with **sC18-SNBD** (10 μM, green) and the lysosomal counterstain LysoTracker (red). **sC18-SNBD** was added, and fluorescence images were immediately recorded. Excitation lasers: 405 nm (for **sC18-SNBD**), 561 nm (for LysoTracker Red). Movie recorded for 20 min and compressed (jpeg) at 10 fps.

Movie S4. Time-lapse fluorescence lifetime imaging of sC18-SNBD. Time-lapse FLIM images of HeLa cells after incubation with **sC18-SNBD** (10 μM). **sC18-SNBD** was added, and FLIM images were immediately recorded. Excitation laser: 440 nm. Movie recorded for 30 min and compressed (jpeg) at 10 fps.

5. NMR Spectra

SNBD-Br (^{1}H NMR: $CDCl_3$; ^{13}C-NMR: $CDCl_3$)

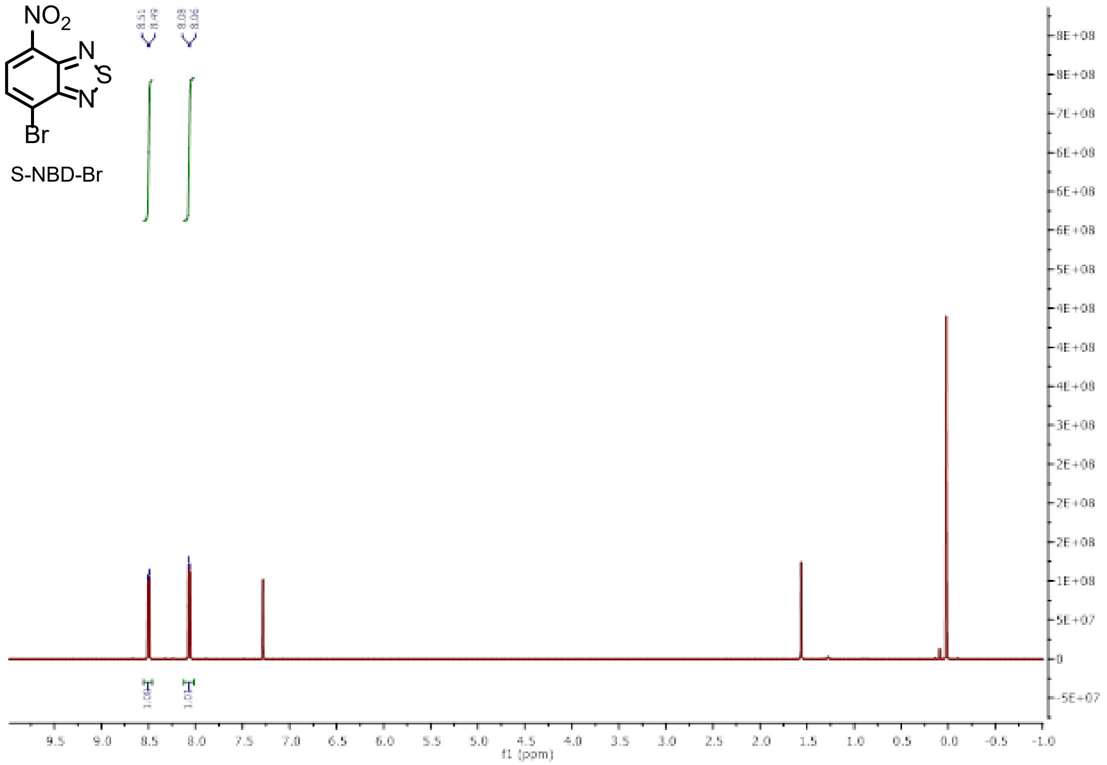

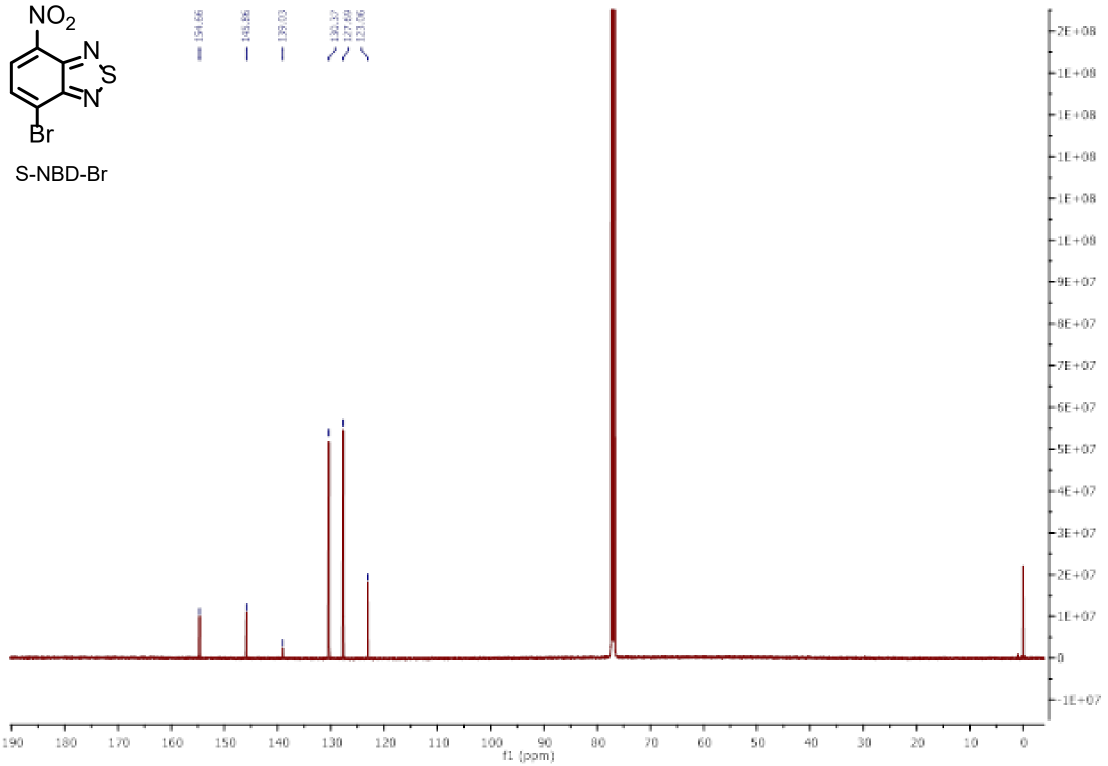

Pd-SNBD (^{1}H NMR: CD_2Cl_2; ^{13}C-NMR: $CDCl_3$; ^{31}P-NMR: $CDCl_3$)

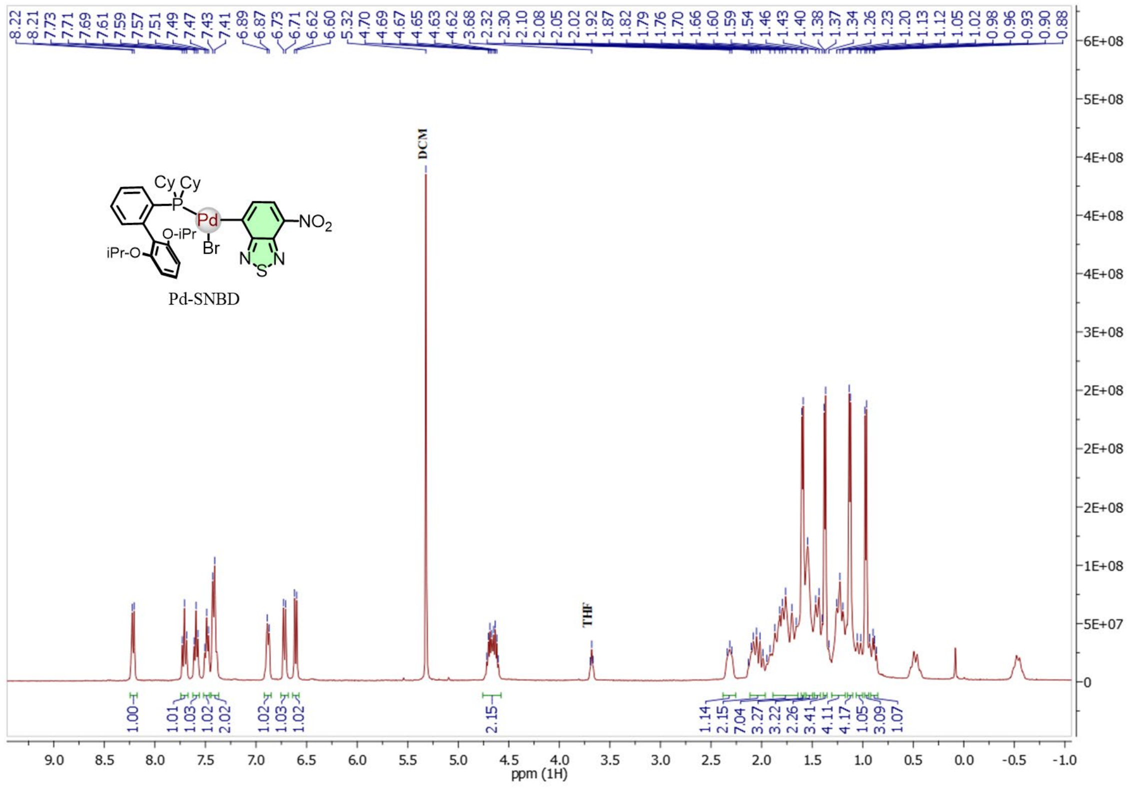

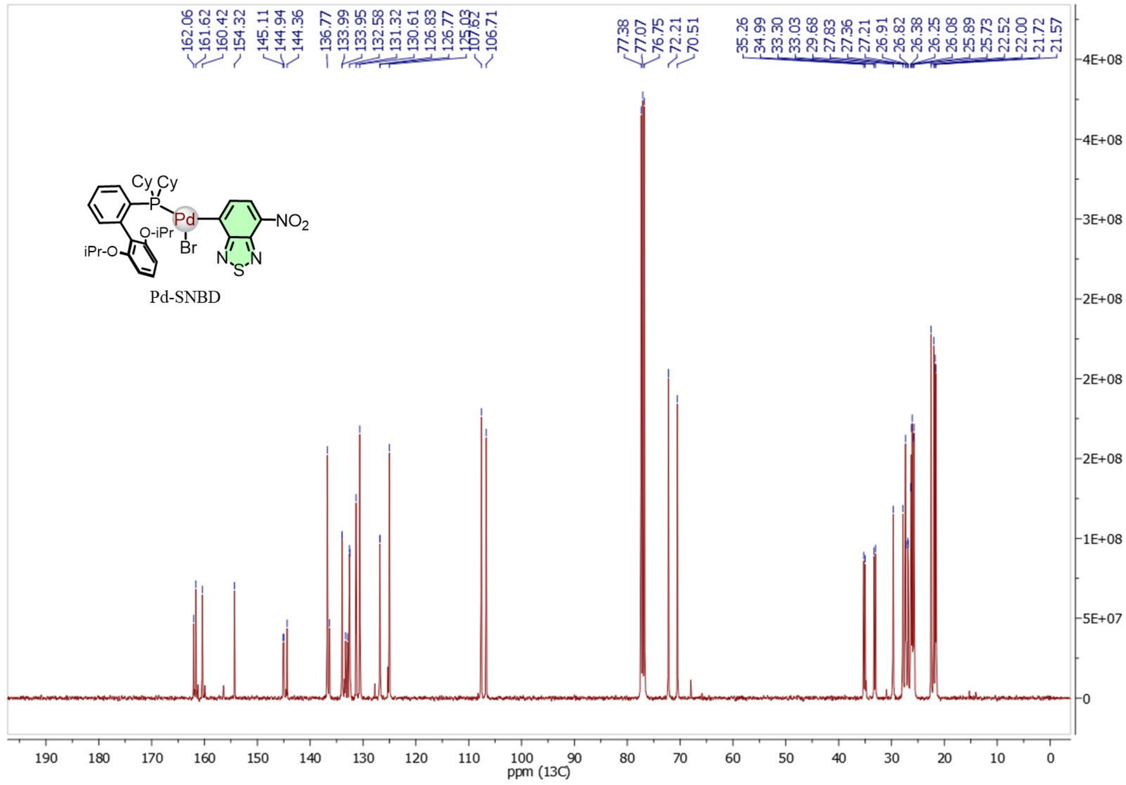

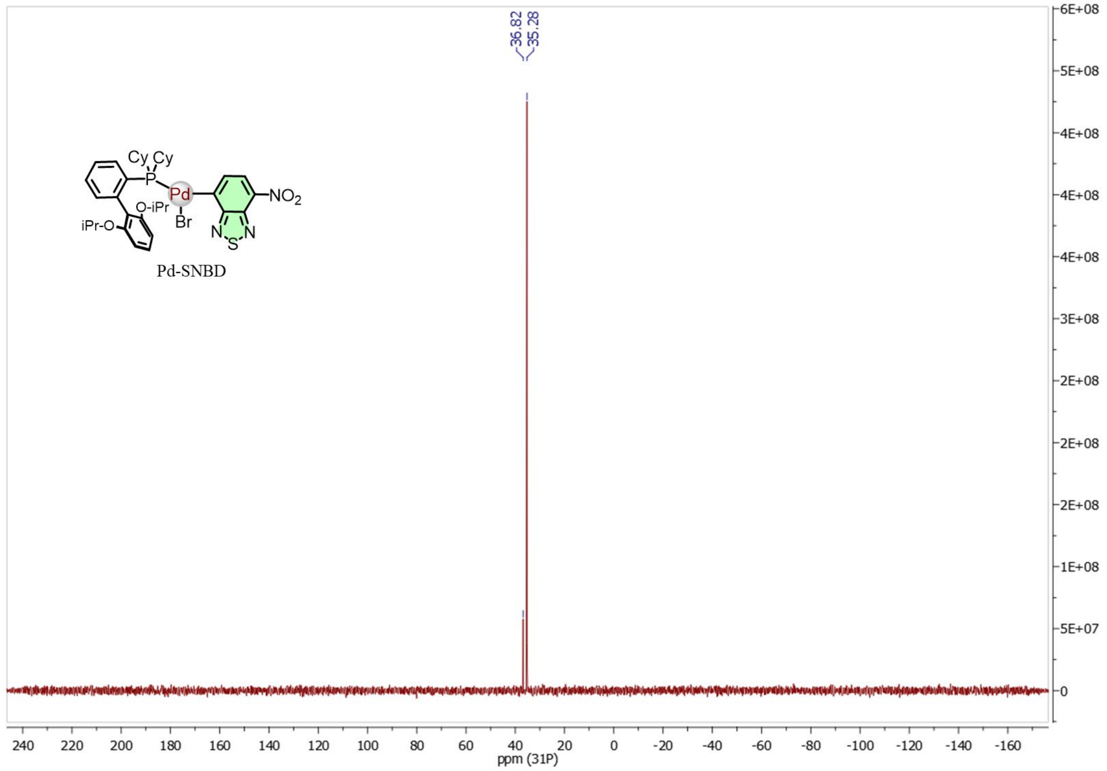
36.82
35.28
Cy Cy
P
Pd
Br
O-iPr
iPr-O
NO2
N
S
N
Pd-SNBD
ppm (31P)

6. Crystal Data and Structure Refinement

Crystals of **Pd-SNBD** were obtained by vapor diffusion of a THF solution of **Pd-SNBD** in pentane at r.t. The molecular structure of **Pd-SNBD** in the solid state was unequivocally determined by single crystal X-ray diffraction analysis. The structure refinement for **Pd-SNBD** complex was obtained as dimer. The crystal structure was uploaded to CCDC with deposition number 2374052.

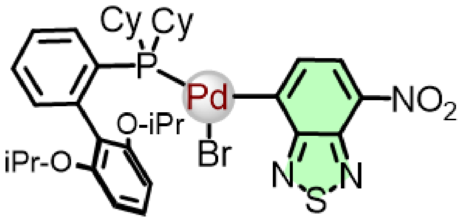

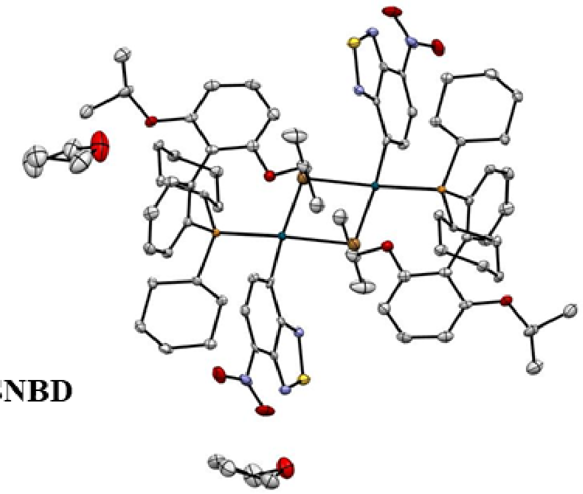

Pd-SNBD

Identification code	jb1
Chemical formula	$C_{88}H_{122}Br_2N_6O_{12}P_2Pd_2S_2$
Formula weight	1954.59 g/mol
Temperature	110(2) K
Wavelength	0.71073 Å
Crystal size	0.047 x 0.100 x 0.147 mm
Crystal habit	yellow prism
Crystal system	triclinic
Space group	P -1

Unit cell dimensions	a = 11.3967(4) Å	α = 66.073(2)°
	b = 14.2075(5) Å	β = 71.078(2)°
	c = 15.6612(6) Å	γ = 81.322(2)°
Volume	2192.11(14) $Å^3$	
Z	1	
Density (calculated)	1.481 g/cm^3	
Absorption coefficient	1.463 mm^{-1}	
F(000)	1012	

Theta range for data collection	1.70 to 27.51°
Index ranges	-14<=h<=14, -18<=k<=18, -20<=l<=20
Reflections collected	67542
Independent reflections	10060 [R(int) = 0.0970]
Coverage of independent reflections	99.6%
Absorption correction	Multi-Scan
Max. and min. transmission	0.9340 and 0.8140
Structure solution technique	direct methods
Structure solution program	SHELXT 2014/5 (Sheldrick, 2014)
Refinement method	Full-matrix least-squares on F^2

Refinement program	SHELXL-2017/1 (Sheldrick, 2017)
Function minimized	$\Sigma\ w(F_o^2 - F_c^2)^2$
Data / restraints / parameters	10060 / 0 / 509
Goodness-of-fit on F^2	1.048
Final R indices	6871 data; $I>2\sigma(I)$ R1 = 0.0486, wR2 = 0.1098
	all data R1 = 0.0926, wR2 = 0.1318
Weighting scheme	$w=1/[\sigma^2(F_o^2)+(0.0341P)^2+12.8434P]$ where $P=(F_o^2+2F_c^2)/3$
Absolute structure parameter	0.00(3)
Largest diff. peak and hole	1.132 and -2.120 eÅ^{-3}
R.M.S. deviation from mean	0.143 eÅ^{-3}

7. Supplementary References

1. De Moliner, F.; Konieczna, Z.; Mendive-Tapia, L.; Saleeb, R. S.; Morris, K.; Gonzalez-Vera, J. A.; Kaizuka, T.; Grant, S. G. N.; Horrocks, M. H.; Vendrell, M. Small Fluorogenic Amino Acids for Peptide-Guided Background-Free Imaging. *Angew. Chem. Int. Ed.* **2023**, *62*, e202216231.

2. Vinogradova, E. V.; Zhang, C.; Spokoyny, A. M.; Pentelute, B. L.; Buchwald, S. L. Organometallic palladium reagents for cysteine bioconjugation. *Nature* **2015,** *526*, 687–691.

3. McAtee, J. R.; Martin, S. E. S.; Ahneman, D. T.; Johnson, K. A.; Watson, D. A. Preparation of Allyl and Vinyl Silanes by the Palladium-Catalyzed Silylation of Terminal Olefins: A Silyl-Heck Reaction. *Angew. Chem. Int. Ed.* **2012**, *51,* 3663–3667.

4. Zheng, J. S.; Tang, S.; Qi, Y. K.; Wang, Z. P.; Liu, L. Chemical synthesis of proteins using peptide hydrazides as thioester surrogates. *Nat. Protoc.* **2013**, *8*, 2483–2495.

5. Dawson, P. E.; Muir, T. W.; Clark-Lewis, I.; Kent, S. B. H. Synthesis of Proteins by Native Chemical Ligation. *Science* **1994**, *266*, 776–779.

6. Lin, X.; Nithun, R. V.; Samanta, R.; Harel, O.; Jbara, M. Enabling Peptide Ligation at Aromatic Junction Mimics via Native Chemical Ligation and Palladium-Mediated S-Arylation. *Org. Lett.* **2023**, *25*, 4715–4719.
