# Supplementary material for: Late-Stage Minimal Labeling of Peptides and Proteins for Real-Time Imaging of Cellular Trafficking
Source: ACS Cent Sci. 2024 Nov 26;11(1):66–75. doi: 10.1021/acscentsci.4c01249 (PMC11758221; doi:10.1021/acscentsci.4c01249)
Supplement: Supplementary file 6 — oc4c01249_si_006.pdf [file oc4c01249_si_006.pdf]

oc-2024-01249m.R1

Name: Peer Review Information for "Late-stage Minimal Labeling of Peptides and Proteins for Real-time Imaging of Cellular Trafficking"

First Round of Reviewer Comments

Reviewer: 1

Comments to the Author

In this manuscript, the authors developed a novel method for fluorescence labeling unprotected peptides and proteins via Pd-mediated arylation. They used benzodiazoles as a small fluorescent molecule (179 Da) for labeling peptides and proteins in which its incorporation should induce little or no effect on the peptide or protein target. In a previous study of the Vendrell group, they developed benzodiazole fluorescent amino acid, yet were unable to incorporate it in peptides using SPPS due to its low stability. Notably, this amino acid has high signal-to-background (S/B) ratios, which enable wash-free microscopy imaging experiments. In this manuscript, the authors modified unprotected peptide to incorporate it on a free Cys residue the benzodiazole via Pd-mediated arylation so called “Late-stage Minimal Labeling of Peptides and Proteins”. The authors discussed the advantages of such a method over the incorporation of benzodiazole via SPPS.

The authors screened the fluorescence emission of 3 different Cys-benzodiazole; Se-bridged, S-bridged, and O-bridged, and have shown that the S-bridged exhibits 27-fold in florescent. The authors clearly and intensively demonstrated the applicability of the method on linear and cyclic peptides, several CPPs, and a small protein (chemokine) which has two disulfide bonds and one free Cys.

Following this, the authors performed intensive microscopy experiments on three reported CPPs; TAT, penetratin, and sC18. They follow the delivery and the internalization mechanism of all three CPPs in live cells with spatiotemporal resolution in a free washing system. The advantage of such a system is the environment fluorescent emission dependence of the benzodiazole, where the fluorescence is dramatically increased in the hydrophobic microenvironment compared to the hydrophilic one. This environment-dependent fluorescence allows the absence of fluorescence in media which enables wash-free experiments for examining the internalization in live time before incubation of the CPPs and examining the delivery mechanism and the final localization. Finally, the authors used the Pd-mediated arylation for modifying a Cys in a small protein that has 2 disulfide bonds. They successfully incorporate the benzodiazoles moiety on additional Cys on the C’ terminal without disrupting the disulfide bonds as was shown in gel analysis and MS spectra. The MS is well-written and easy to follow with excellent data on the chemistry and microscopy parts. I recommend publication with a minor revision, as listed below:

1- In line 58 the authors use the term “SNBD” without defining it neither in the abstract nor the introduction.

2- In Figure 1.b the dioxane: water heat map key is confusing for the reader; the authors should emphasize which color corresponds to which environment.

3- In Figure 1.b the authors showed fluorescent emission ratio (6-fold, 27-fold, and 10-fold) it is unclear, on what basis they made their conclusions.

4- In Figure 6.b the Coomassie gel showed two bands for the mCCL2. However, when the protein was modified with the benzodiazoles using palladium the gel showed one band. Based on the MS spectra it’s clear that the free C’ terminal Cys was modified without disrupting the disulfide bond. Can the authors explain this?

5- The authors should be consistent with the name of the benzodiazole, as sometimes written as nitro- benzodiazole.

6- The CIF file of the reported X-ray structure [Pd-SNBD complex] is missing. The authors must upload it.

Reviewer: 2

Comments to the Author

In this manuscript the groups of Jbara and Vendrell report about the synthesis of Pd-complexes of the Thia-analog (“SNBD”) of the widely used fluorescence dye NBD (4-nitro-2,1,3-benzoxadiazole) and its use for labelling via a Buchwald-Pentelute type S-arylation of Cys in small peptides and a small protein (mouse chemokine ligand 2 (mCCL2)). The labelling is advertised as a method which is distinguished by its efficiency and simplicity.

The SNBD chromophore (and other chalcogeno analogs) have already been described in peptides by the same authors in a recent Angew. Chem. publication (2023), where they have been introduced as amino acid building blocks via SPPS.

The novelty of the current manuscript lies in the “late-stage” introduction of the fluorescent dye via the Pd-mediated reaction, and the use of the synthesized peptide probes in studying its transport across membranes.

I like the presented research, but in my personal opinion it is not of sufficient originality and novelty to be published in ACS Cent. Sci, but rather recommend ACS Chem. Biol. instead.

Before publication the following issues need to be addressed:

1) It is not clear from the manuscript, why the authors used the Pd-mediated labelling and not an SNar-type labelling as it is commonly used for NBD-Cl with thiols or other nucleophiles.

This could overcome the limitation that their Pd-SNBD complex can only be produced in small amounts (up to 50 mg), which is relevant since only a minor share of the reagent represents the fluorescent dye.

2) I missed in the manuscript a full characterization of the labelled peptides. From the provided data it cannot be concluded that the labelling occurred on Cys (although I agree that this would be very likely) or any other of the nucleophilic amino acids.

Did the authors check if their purified peptides contain any remaining Pd impurities? This could be relevant for their applications in cell biology, as it is associated with a certain level of toxicity.

3) A continuous theme of the manuscript is the advertisement of the "minimal" character of these dyes. However, this is well established for the many applications of NBD-labelled proteins and peptides, as has been summarized in the following review (which should also be cited in the revised manuscript):

NBD-based synthetic probes for sensing small molecules and proteins: design, sensing mechanisms and biological applications

Chenyang Jiang, Haojie Huang, Xueying Kang, Liu Yang, Zhen Xi, Hongyan Sun, Michael D. Pluth, Long Yi

Chem. Soc. Rev., 2021, 50, 7436

DOI: 10.1039/d0cs01096k

4) I had difficulties to understand what is meant with "O-bridged", "S-bridged", "Se-bridged", as I have first thought that it refers to the linkage of the fluorophore to the peptide and later got the impression that it refers to the chalcogeno atom in the flurophore.

I would recommend that the authors use the systematic names, e.g. benzo-2,1,3-thiadiazole to avoid any misunderstanding.

In conclusion, I can recommend the publication of this manuscript in ACS Chem. Biol, once all the issues raised in this report will have been addressed.

Minor comments:

throughout the manuscript: e.g. 25 °C instead of 25°C

p.5.line 5: synthesized instead of synthetized

p.10. line 36: “calculated logP (clogP)” instead of “coefficient logP (clogP)”

Reviewer: 3

Comments to the Author

Authors report an elegant study translating Pd-based bioconjugation towards modification of functional peptides and proteins for imaging. I think this work represents an elegant marriage of several fields and is really well executed on a technical level. Publication is recommended after the following minor comments that I encourage authors to address:

Numerous cyclic peptides are cyclized via the disulfide bonds. Does this chemistry operate on a macrocycle as such (i.e., unfunctionalized Cys residue on a macrocyclic peptide with a disulfide bond)?

How general do authors think the Pd chemistry is for other scaffolds Vendrell and co-workers have developed (including some mentioned in this work)?

Authors should demonstrate and quantify the removal of Pd-based content from their reactions considering they do in cellulo experiments.

For the workflow in Figure 3, can authors comment why the elected to embed their Cys residue at the C terminus as opposed to N? I also think there is a slight disconnect between the model studies where authors should modification of Cys residues embededed in the polypeptide sequence and the subsequent functional studies that focus on the terminal Cys modification.

Author's Response to Peer Review Comments:

Formatting Needs

SYNOPSIS: The synopsis should be no more than 200 characters (including spaces) and should reasonably correlate with the TOC graphic. The synopsis is intended to explain the importance of the article to a broader readership across the sciences. Please place your synopsis in the manuscript file after the TOC graphic, and label it as "Synopsis." **Response:** We have amended the synopsis.

SI HEADER: The title and author list on the first page of the SI for Pub file must match the title and author list of the manuscript.

Response: We have corrected the title on the first page of the Supporting Information and included the author list.

Response to Reviewers Reviewer 1

Comment 1. *In line 58 the authors use the term "SNBD" without defining it neither in the abstract nor the introduction.*

Response: We have defined the terms benzo-2,1,3-thiadiazole (SNBD), as well as benzo2,1,3-oxadiazole (ONBD) and benzo-2,1,3-selenadiazole (SeNBD). Page 5, Paragraph 1.

Comment 2*. In Figure 1.b the dioxane: water heat map key is confusing for the reader; the authors should emphasize which color corresponds to which environment.*

Response: We have edited the legend of Figure 1B to clarify how the heat map key corresponds to high and low dioxane:water ratio mixtures.

Comment 3. *In Figure 1.b the authors showed fluorescent emission ratio (6-fold, 27-fold, and 10-fold) it is unclear, on what basis they made their conclusions.*

Response: The ratios were calculated by dividing the emission intensities of each fluorophores in 8:2 and 1:9 dioxane:water mixtures. We have clarified this point in Figure 1.

Comment 4. *In Figure 6.b the Coomassie gel showed two bands for the mCCL2. However, when the protein was modified with the benzodiazoles using palladium the gel showed one band. Based on the MS spectra it's clear that the free C' terminal Cys was modified without disrupting the disulfide bond. Can the authors explain this?*

Response: Thank you for this observation. The band observed for unlabeled mCCL2 corresponds to the dimer (~20 kDa) formed via disulfide bonds involving the free C-terminal Cys residue (references 63 and 64 in the manuscript). On the other hand, the reaction between mCCL2 and **Pd-SNBD** results in the formation of mCCL2-SNBD with a single SNBD transfer to the monomeric mCCL2, as confirmed via LC-MS and in-gel fluorescence scanning (Figure

6).

Comment 5. *The authors should be consistent with the name of the benzodiazole, as sometimes written as nitro-benzodiazole.*

Response: We have amended the nomenclature to use 'benzodiazole' consistently throughout the manuscript.

Comment 6. *The CIF file of the reported X-ray structure [Pd-SNBD complex] is missing. The authors must upload it.*

Response: We have uploaded the CIF file corresponding to the X-ray structure of **PD-SNBD** [CCDC: 2374052].

Reviewer 2

Comment 1. *It is not clear from the manuscript, why the authors used the Pd-mediated labelling and not an SNar-type labelling as it is commonly used for NBD-Cl with thiols or other nucleophiles. This could overcome the limitation that their Pd-SNBD complex can only be produced in small amounts (up to 50 mg), which is relevant since only a minor share of the reagent represents the fluorescent dye.*

Response: We chose Pd-mediated S-arylation due to its robust reactivity and selectivity for Cys under biocompatible conditions, as demonstrated in this work with the modification of unprotected peptides and proteins with rapid and high-yielding outcomes. The power of Pdmediated S-arylation chemistry is highlighted in the introduction, where we have included several references on the topic. Regarding the scale, the protocol for labeling proteins requires only micrograms of the **Pd-SNBD** complex; therefore, the synthesis of **Pd-SNBD** in milligrams scale is sufficient for dozens of protein analogs.

Comment 2. *I missed in the manuscript a full characterization of the labelled peptides. From the provided data it cannot be concluded that the labelling occurred on Cys (although I agree that this would be very likely) or any other of the nucleophilic amino acids. Did the authors check if their purified peptides contain any remaining Pd impurities? This could be relevant for their applications in cell biology, as it is associated with a certain level of toxicity.*

Response: We thank the reviewer for the comment. We have confirmed the reactivity of **PdSNBD** with Cys by LC-MS/MS (new Figure S4) and using an analogue of **P1** that does not contain Cys residues as a negative control (new Figure S5). To measure any potential residual Pd in isolated peptides, we determined the Pd content in the purified **penetratin-SNBD** using inductively coupled plasma mass spectrometry (ICP-MS), which was found to be 15 ppm. This amount of Pd poses no toxicity for cellular studies (Page 8, Paragraph 2).

Comment 3. *A continuous theme of the manuscript is the advertisement of the "minimal" character of these dyes. However, this is well established for the many applications of NBDlabelled proteins and peptides, as has been summarized in the following review (which should also be cited in the revised manuscript): NBD-based synthetic probes for sensing small molecules and proteins: design, sensing mechanisms and biological applicationsChenyang Jiang, Haojie Huang, Xueying Kang, Liu Yang, Zhen Xi, Hongyan Sun, Michael D. Pluth, Long Yi Chem. Soc. Rev., 2021, 50, 7436 DOI: 10.1039/d0cs01096k*

Response: Thank you for the comment. We have now included the reference in the revised manuscript (reference 30).

Comment 4. *I had difficulties to understand what is meant with "O-bridged", "S-bridged", "Sebridged", as I have first thought that it refers to the linkage of the fluorophore to the peptide*

and later got the impression that it refers to the chalcogeno atom in the flurophore. I would recommend that the authors use the systematic names, e.g. benzo-2,1,3-thiadiazole to avoid any misunderstanding.

Response: As mentioned above, we have amended the nomenclature and referred the compounds as benzo-2,1,3-oxadiazole (ONBD), benzo-2,1,3-thiadiazole (SNBD), and benzo2,1,3-selenadiazole (SeNBD).

Comment 5. *Throughout the manuscript: e.g. 25 °C instead of 25°C* **Response:** Corrected in the revised manuscript.

Comment 6. *p.5.line 5: synthesized instead of synthetized* **Response:** Corrected in the revised manuscript.

Comment 7. *p.10. line 36: "calculated logP (clogP)" instead of "coefficient logP (clogP)"* **Response:** Corrected in the revised manuscript.

Reviewer 3

Comment 1. *Numerous cyclic peptides are cyclized via the disulfide bonds. Does this chemistry operate on a macrocycle as such (i.e., unfunctionalized Cys residue on a macrocyclic peptide with a disulfide bond)?*

Response: We thank the reviewer for this comment. We appreciate that cyclization via disulfide bonds is a popular strategy to enhance peptide and protein properties, such as stability and cell penetration. This rationale influenced our selection of mCCL2 as a model protein, as it contains two disulfide bonds (Figure 6A). Labeling of mCCL2 under non-reducing conditions left the disulfide bonds unaffected, as confirmed by SDS-PAGE gel and LC-MS (Figure 6C). These results demonstrate that the reported labeling strategy is compatible with peptides containing disulfide bonds. Furthermore, we showed that our strategy can be applied to head-to-tail cyclic peptides, as demonstrated with **cP1** (Figure 2). Given the importance of this point, we have clarified the presence of disulfide bonds in mCCL2 and their compatibility with our labeling approach in the revised manuscript (Page 15, Paragraph 1, Figure 6).

Comment 2. *How general do authors think the Pd chemistry is for other scaffolds Vendrell and co-workers have developed (including some mentioned in this work)?*

Response: This chemistry could be applied in principle to other fluorophores, which will be reported in due course. Their feasibility will likely depend on the reactivity of the fluorophores and should be evaluated on a case-by-case basis.

Comment 3. *Authors should demonstrate and quantify the removal of Pd-based content from their reactions considering they do in cellulo experiments.*

Response: This comment was also raised by reviewer 2. To measure any potential residual Pd in isolated peptides, we determined the Pd content in the purified **penetratin-SNBD** using ICP-MS, which was found to be 15 ppm. This amount of Pd poses no toxicity for cellular studies (Page 8, Paragraph 2).

Comment 4. *For the workflow in Figure 3, can authors comment why the elected to embed their Cys residue at the C terminus as opposed to N? I also think there is a slight disconnect between the model studies where authors should modification of Cys residues embededed in the polypeptide sequence and the subsequent functional studies that focus on the terminal Cys modification.*

Response: We thank the reviewer for the comment. The arylation of Cys residues with Pd complexes can be used to functionalize proteins regardless of the site in the sequence (e.g., the N-terminus, C-terminus, or internal position; see reference 38). In this work, we initially attempted the arylation of peptides using the new **Pd-SNBD** reagent on internal Cys residues to confirm that surrounding functional groups did not interfere with the S-arylation reaction. For the labeling of CPPs and mCCL2, we incorporated the Cys residue at the C-terminal end to minimize any potential

perturbation of their bioactivity. The C-terminal end was chosen because the N-terminus of mCCL2 is essential for receptor binding. We have clarified this point in the revised manuscript.

oc-2024-01249m.R2

Name: Peer Review Information for "Late-stage Minimal Labeling of Peptides and Proteins for Real-time Imaging of Cellular Trafficking"

Second Round of Reviewer Comments

Reviewer: 2

Comments to the Author

In this revised version the authors have addressed all issues raised by the reviewers of the previous manuscript. As former reviewer #2 I am particular happy that additional data now demonstrate that labelling has occured at Cys and that the amount of residual Pd is rather low.

I now recomment the publication of this manuscript as it is.

Reviewer: 1

Comments to the Author

The authors have carefully and fully addressed all comments for my comments and others. I want to congratulate them for this inspiring study.

Reviewer: 3

Comments to the Author

Authors have addressed my comments, publication is now recommended.

Author's Response to Peer Review Comments:

Dear Editor,

Thank you for the positive response. We are uploading the final files after addressing the editorial requests.

Sincerely,

Marc Vendrell
